# Supplementary material for: A systematic review of non-clinician trauma-based interventions for school-age youth
Source: PLoS One. 2024 Sep 6;19(9):e0293248. doi: 10.1371/journal.pone.0293248 (PMC11379276; doi:10.1371/journal.pone.0293248)
Supplement: S1 Table — (DOCX) [file pone.0293248.s001.docx]

# **Supporting information 1. Quality Assessment Tables**

### Table 2. Quality of included RCTs

| First author (year) | Selection bias rating | Study design rating | Confounders rating | Blinding rating | Data Collection Rating | Dropouts and Withdrawals Rating | Final rating |
| --- | --- | --- | --- | --- | --- | --- | --- |
| Akhtar (2019) | 1 | 1 | 3 | 2 | 3 | 3 | Weak (two or more weak ratings) |
| Barron (2021) | 3 | 2 | 1 | 2 | 1 | 2 | Moderate (one weak rating) |
| Bryant (2022) | 2 | 1 | 1 | 2 | 3 | 1 | Moderate (one weak rating) |
| El-Khani (2021) | 1 | 1 | 1 | 2 | 1 | 1 | Strong (no weak ratings) |
| Goldbach (2021) | 2 | 2 | 1 | 2 | 3 | 1 | Moderate (one weak rating) |
| Li (2023) | 2 | 1 | 1 | 2 | 3 | 3 | Weak (two or more weak ratings) |

Assessed using the Effective Public Health Practice Project (EPHPP)’s Quality Assessment Tool for Quantitative Studies. 1 is the strongest rating and 3 is the weakest.

### Table 3. Quality of cohort design studies

| First author (year) | Selection bias rating | Study design rating | Confounders rating | Blinding rating | Data Collection Rating | Dropouts and Withdrawals Rating | Final rating |
| --- | --- | --- | --- | --- | --- | --- | --- |
| Barnett (2020) | 2 | 2 | 3 | 2 | 3 | 1 | Weak (2 or more weak ratings) |
| Davis (2022) | 3 | 2 | 3 | 2 | 1 | 3 | Weak (2 or more weak ratings) |
| Day (2015) | 3 | 2 | 3 | 2 | 3 | 3 | Weak (2 or more weak ratings) |
| Dumornay (2022) | 3 | 2 | 3 | 2 | 3 | 3 | Weak (2 or more weak ratings) |
| El-Khani (2018) | 3 | 2 | 3 | 2 | 1 | 1 | Weak (2 or more weak ratings) |
| Elswick (2022) | 2 | 2 | 3 | 2 | 3 | 3 | Weak (2 or more weak ratings) |
| Eruyar (2020) | 2 | 2 | 1 | 2 | 3 | 3 | Weak (2 or more weak ratings) |
| Greenbaum (2017) | 2 | 1 | 1 | 2 | 2 | 1 | Strong (no weak ratings) |
| Ito (2021) | 2 | 2 | 3 | 2 | 1 | 1 | Moderate (one weak rating) |
| Jaycox (2019) | 2 | 2 | 3 | 2 | 3 | 3 | Weak (2 or more weak ratings) |
| Martin (2017) | 3 | 2 | 3 | 2 | 1 | 3 | Weak (2 or more weak ratings) |
| Murray (2013) | 2 | 2 | 3 | 2 | 2 | 2 | Moderate (one weak rating) |
| Sandhu (2016) | 2 | 2 | 3 | 2 | 1 | 3 | Weak (2 or more weak ratings) |
| Sarkadi (2018)* | 2 | 2 | 3 | 2 | 3 | 3 | Weak (2 or more weak ratings) |
| Schuurmans (2020) | 2 | 2 | 3 | 2 | 1 | 2 | Moderate (one weak rating) |
| Sitzer (2015) | 2 | 2 | 3 | 2 | 3 | 3 | Weak (2 or more weak ratings) |
| Taku (2017) | 2 | 2 | 1 | 2 | 3 | 1 | Moderate (one weak rating) |

*included in Tables 3 and 4 as quantitative and qualitative aspects were assessed separately
Assessed using the Effective Public Health Practice Project (EPHPP)’s Quality Assessment Tool for Quantitative Studies. 1 is the strongest rating and 3 is the weakest.

### Table 4. Quality of qualitative studies

| First author (year) | Clearly stated aim? | Qualitative methodology appropriate? | Appropriate research design to address the aims of the research? | Appropriate recruitment strategy to address the aims of the research? | Was the data collected in a way that addressed the research issue? | Has the relationship between researcher and participants been adequately considered? | Have ethical issues been taken into consideration? | Was the data analysis rigorous? | Is there a clear statement of findings? |
| --- | --- | --- | --- | --- | --- | --- | --- | --- | --- |
| Harden (2015) | Yes | Can't tell | Can't tell | No | Can't tell | No | Can't tell | Can't tell | Can't tell |
| McMahon (2020) | Yes | Yes | Yes | No | No | No | No | No | Yes |
| Özden Bademci (2015) | Yes | Yes | Yes | No | Can't tell | No | Can't tell | Yes | Can't tell |
| Sarkadi (2018) | Yes | Yes | Yes | Yes | Yes | No | Yes | Yes | Yes |

Assessed using the Critical Appraisal Skills Programme (CASP) Qualitative Studies Checklist
