# Supplementary material for: A systematic review of non-clinician trauma-based interventions for school-age youth
Source: PLoS One. 2024 Sep 6;19(9):e0293248. doi: 10.1371/journal.pone.0293248 (PMC11379276; doi:10.1371/journal.pone.0293248)
Supplement: S1 File — (DOCX) [file pone.0293248.s002.docx]

**Supporting information 2. Search strategy**

The following table details the search strategy used for this systematic review.

A 10 year date limit was imposed (e.g since January 2013).

Only English language studies were included.

| **Database** | **Date** | **Search strategy** | **Number of results** |
| --- | --- | --- | --- |
| Web of Science | 5 April 2023 | 1. Trauma* OR "Post-Traumatic Stress" OR PTSD (Topic)  2. and Intervention* OR Treatment* (Topic)  3. and children OR youth OR young OR adolescen* (Topic)  4. and education OR school OR teach* OR play (Topic)  5. not review OR meta-analysis OR preschool OR pediatric OR longitudinal OR "very young children" OR toddlers (Topic)  Timespan: 2013-01-01 to 2023-04-04 (Index Date)  Query link: <https://www.webofscience.com/wos/woscc/summary/9fbb7d01-4872-4f9e-8db4-c403e1f3eb65-7f295b61/recently-added/1> | 2105 |
| EBSCO (CINAHL Plus with Full Text, MEDLINE, APA PsycArticles, APA PsycInfo, Teacher Reference Center, Education Research Complete) | 5 April 2023 | ( Trauma* OR "Post-Traumatic Stress" OR PTSD ) AND ( Intervention* OR Treatment* ) AND ( children OR youth OR young OR adolescen* ) AND ( education OR school OR teach* OR play ) NOT ( not review OR meta-analysis OR preschool OR pediatric OR longitudinal OR "very young children" OR toddlers )  Limiters - Full Text; Peer Reviewed; Publication Date: 20130101-  Search modes - Boolean/Phrase | 2,458 imported  (1889 duplicates)  569 imported |
| EMBASE | 25 April 2023 | (**trauma***:ti,ab,kw OR **'post-traumatic stress'**:ti,ab,kw OR **'posttraumatic stress disorder'**:ti,ab,kw) AND (**intervention***:ti,ab,kw OR **treatment**:ti,ab,kw) AND (**children**:ti,ab,kw OR **youth**:ti,ab,kw OR **young**:ti,ab,kw OR **adolescent**:ti,ab,kw) AND (**education**:ti,ab,kw OR **school**:ti,ab,kw OR **teach***:ti,ab,kw OR **play**:ti,ab,kw) NOT (**review**:ti,ab,kw OR **'meta analysis'**:ti,ab,kw OR **preschool**:ti,ab,kw OR **pediatric**:ti,ab,kw OR **longitudinal**:ti,ab,kw OR **'very young children'**:ti,ab,kw OR **toddler**:ti,ab,kw) AND [2013-2023]/py AND ([adolescent]/lim OR [adult]/lim OR [preschool]/lim OR [school]/lim) AND [english]/lim | 1,132 imported  (608 duplicates)  524 imported |
| ASSIA | 25 April 2023 | noft(Trauma* OR "Post-Traumatic Stress" OR PTSD) AND noft(Intervention* OR Treatment*) AND noft(children OR youth OR young OR adolescen*) AND noft(education OR school OR teach* OR play) NOT noft(review OR meta-analysis OR preschool OR pediatric OR longitudinal OR "very young children" OR toddlers OR dental) | 918 imported  (377 duplicates)  541 imported |
| Science Direct | 25 April 2023 | Title, abstract or author-specified keywords: (Trauma OR "Post-Traumatic Stress" OR PTSD) AND (Intervention OR Treatment) AND (children OR youth OR young OR adolescent)  Title:  NOT (review OR meta-analysis OR preschool OR pediatric OR longitudinal OR "very young children" OR toddlers)  Subject areas:  Medicine and Dentistry (534)  Psychology (416)  Social Sciences (346)  Neuroscience (49)  Nursing and Health Professions  Publication title:  Child Abuse & Neglect (173)  Children and Youth Services Review (136)  Journal of Affective Disorders (46)  Journal of the American Academy of Child & Adolescent Psychiatry (43)  European Psychiatry (30)  Journal of Psychiatric Research (25)  Psychiatry Research (24)  Journal of Adolescent Health (22)  Child and Adolescent Psychiatric Clinics of North America (20)  The Arts in Psychotherapy (15) | 534 imported  (133 duplicates)  401 imported |
