# Supplementary material for: A systematic review of non-clinician trauma-based interventions for school-age youth
Source: PLoS One. 2024 Sep 6;19(9):e0293248. doi: 10.1371/journal.pone.0293248 (PMC11379276; doi:10.1371/journal.pone.0293248)
Supplement: S2 File — (DOCX) [file pone.0293248.s003.docx]

## Supporting information 3. Data Extraction Headings

Study ID

Was the study population specifically high ACEs?

How were ACEs measured?

How were PTSD/trauma symptoms measured?

PTSD Scores

Above clinical threshold?

PTSD baseline

PTSD post score

Significance

Quality assessment

Outcome measures

What was the outcome?

Interpretation

Tickbox headings:

Group

Individual

Curriculum/whole school

With caregivers

Art

Music

CBT

Movement

Mindfulness

Mentoring

Youth led

Restorative justice

Writing

Emotional regulation

In School

At University

Residential

Community

Various

Online

Primary age

Secondary age

Post-16
