# Supplementary material for: A systematic review of non-clinician trauma-based interventions for school-age youth
Source: PLoS One. 2024 Sep 6;19(9):e0293248. doi: 10.1371/journal.pone.0293248 (PMC11379276; doi:10.1371/journal.pone.0293248)
Supplement: Attachment — Submitted filename: Feedback MAKHADO PONE-D-23-32516.pdf [file pone.0293248.s005.pdf]

PLOS ONE

A systematic review of non-clinician trauma-based interventions for school-age children and young people

--Manuscript Draft--

**Manuscript Number:**	PONE-D-23-32516
**Article Type:**	Research Article
**Full Title:**	A systematic review of non-clinician trauma-based interventions for school-age children and young people
**Short Title:**	Review of non-clinical trauma-based interventions for school children.
**Corresponding Author:**	Sinead Brophy, PhD<br>Swansea University College of Medicine: Swansea University Medical School<br>Swansea, Wales UNITED KINGDOM
**Keywords:**	Trauma-based intervention, school children, non-clinician intervention, Adverse Childhood Events
**Abstract:**	Exposure to adverse childhood experiences (ACEs) is increasingly recognised as a risk factor for health problems in later life. Awareness of ACEs and associated trauma is increasing in schools and educational settings, as well as demand for supportive services to deal with needs. However, there is a lack of clear evidence for effective interventions which can be delivered by non-clinicians (e.g., the school staff themselves). For this review, we conducted a search across five electronic databases for studies published since January 2013. These studies assessed the efficacy of non-clinician delivered interventions for supporting trauma recovery or improvements in mental health in school age youth (4-18 years) who have experienced ACEs. Out of the 4097 studies identified through the search, 326 were retrieved for full text screening and 25 were included in the final review. There was considerable heterogeneity in study design, outcome measures and the intervention being studied. The majority of studies were assessed to be of weak quality due to convenience sampling of participants and potential bias, indicating there is a lack of high quality research evidence to inform non-clinician delivered trauma-informed interventions for improving mental health outcomes in school-age young people. Cognitive Behavioural Therapy (CBT)-based approaches are tentatively suggested as a suitable target for future rigorous evaluations of interventions addressing ACE-related trauma recovery and mental health improvement in school-age young people.
**Order of Authors:**	Flo Avery
	Natasha Kennedy
	Michaela James
	Hope Jones
	Rebekah Amos
	Mark Bellis
	Karen Hughes
	Sinead Brophy, PhD
**Additional Information:**	
**Question**	**Response**
**Financial Disclosure**<br><br>Enter a financial disclosure statement that describes the sources of funding for the work included in this submission. Review the submission guidelines for detailed	Yes

Powered by Editorial Manager® and ProduXion Manager® from Aries Systems Corporation

<table>
<tr><td>requirements. View published research articles from PLOS ONE for specific examples.

This statement is required for submission and will appear in the published article if the submission is accepted. Please make sure it is accurate.

Funded studies
Enter a statement with the following details:
• Initials of the authors who received each award
• Grant numbers awarded to each author
• The full name of each funder
• URL of each funder website
• Did the sponsors or funders play any role in the study design, data collection and analysis, decision to publish, or preparation of the manuscript?

Did you receive funding for this work?</td><td></td></tr>
<tr><td>Please add funding details.
as follow-up to "Financial Disclosure

Enter a financial disclosure statement that describes the sources of funding for the work included in this submission. Review the submission guidelines for detailed requirements. View published research articles from PLOS ONE for specific examples.

This statement is required for submission and will appear in the published article if the submission is accepted. Please make sure it is accurate.</td><td>This work was supported by the National Centre for Population Health and Wellbeing Research. Funding was provided by Public Health Wales and the Economic and Social Research Council (ESRC) through their suport of a PhD studentship</td></tr>
</table>

**Funded studies**<br>Enter a statement with the following details:<br>• Initials of the authors who received each award<br>• Grant numbers awarded to each author<br>• The full name of each funder<br>• URL of each funder website<br>• Did the sponsors or funders play any role in the study design, data collection and analysis, decision to publish, or preparation of the manuscript?<br><br>Did you receive funding for this work?"	
Please select the country of your main research funder (please select carefully as in some cases this is used in fee calculation).<br>as follow-up to "**Financial Disclosure**<br><br>Enter a financial disclosure statement that describes the sources of funding for the work included in this submission. Review the submission guidelines for detailed requirements. View published research articles from *PLOS ONE* for specific examples.<br><br>This statement is required for submission and **will appear in the published article** if the submission is accepted. Please make sure it is accurate.<br><br>**Funded studies**<br>Enter a statement with the following details:<br>• Initials of the authors who received each award<br>• Grant numbers awarded to each author<br>• The full name of each funder<br>• URL of each funder website<br>• Did the sponsors or funders play any role in the study design, data collection and analysis, decision to publish, or preparation of the manuscript?<br><br>Did you receive funding for this work?"	UNITED KINGDOM - GB
**Competing Interests**	The authors have declared that no competing interests exist.

Use the instructions below to enter a competing interest statement for this submission. On behalf of all authors, disclose any competing interests that could be perceived to bias this work—acknowledging all financial support and any other relevant financial or non-financial competing interests.<br><br>This statement is required for submission and **will appear in the published article** if the submission is accepted. Please make sure it is accurate and that any funding sources listed in your Funding Information later in the submission form are also declared in your Financial Disclosure statement.<br><br>View published research articles from *PLOS ONE* for specific examples.<br><br>**NO authors have competing interests**<br><br>Enter: *The authors have declared that no competing interests exist.*<br><br>**Authors with competing interests**<br><br>Enter competing interest details beginning with this statement:<br><br>*I have read the journal's policy and the authors of this manuscript have the following competing interests: [insert competing interests here]*<br><br>* typeset	
**Ethics Statement**<br><br>Enter an ethics statement for this submission. This statement is required if the study involved:<br><br>• Human participants<br>• Human specimens or tissue<br>• Vertebrate animals or cephalopods<br>• Vertebrate embryos or tissues	N/A

• Field research<br><br>Write "N/A" if the submission does not require an ethics statement.<br><br>General guidance is provided below. Consult the submission guidelines for detailed instructions. **Make sure that all information entered here is included in the Methods section of the manuscript.**	

**Format for specific study types**<br><br>**Human Subject Research (involving human participants and/or tissue)**<br>• Give the name of the institutional review board or ethics committee that approved the study<br>• Include the approval number and/or a statement indicating approval of this research<br>• Indicate the form of consent obtained (written/oral) or the reason that consent was not obtained (e.g. the data were analyzed anonymously)<br><br>**Animal Research (involving vertebrate animals, embryos or tissues)**<br>• Provide the name of the Institutional Animal Care and Use Committee (IACUC) or other relevant ethics board that reviewed the study protocol, and indicate whether they approved this research or granted a formal waiver of ethical approval<br>• Include an approval number if one was obtained<br>• If the study involved *non-human primates*, add *additional details* about animal welfare and steps taken to ameliorate suffering<br>• If anesthesia, euthanasia, or any kind of animal sacrifice is part of the study, include briefly which substances and/or methods were applied<br><br>**Field Research**<br><br>Include the following details if this study involves the collection of plant, animal, or other materials from a natural setting:<br>• Field permit number<br>• Name of the institution or relevant body that granted permission	
**Data Availability**<br><br>Authors are required to make all data underlying the findings described fully available, without restriction, and from the time of publication. PLOS allows rare exceptions to address legal and ethical concerns. See the PLOS Data Policy and FAQ for detailed information.	Yes - all data are fully available without restriction

Powered by Editorial Manager® and ProduXion Manager® from Aries Systems Corporation

A Data Availability Statement describing where the data can be found is required at submission. Your answers to this question constitute the Data Availability Statement and **will be published in the article**, if accepted.<br><br>**Important:** Stating 'data available on request from the author' is not sufficient. If your data are only available upon request, select 'No' for the first question and explain your exceptional situation in the text box.<br><br>Do the authors confirm that all data underlying the findings described in their manuscript are fully available without restriction?	
**Describe where the data may be found in full sentences. If you are copying our sample text, replace any instances of XXX with the appropriate details.**<br><br>• If the data are **held or will be held in a public repository**, include URLs, accession numbers or DOIs. If this information will only be available after acceptance, indicate this by ticking the box below. For example: *All XXX files are available from the XXX database (accession number(s) XXX, XXX.*).<br>• If the data are all contained **within the manuscript and/or Supporting Information files**, enter the following: *All relevant data are within the manuscript and its Supporting Information files.*<br>• If neither of these applies but you are able to provide **details of access elsewhere**, with or without limitations, please do so. For example:<br><br>*Data cannot be shared publicly because of [XXX]. Data are available from the XXX Institutional Data Access / Ethics Committee (contact via XXX) for researchers who meet the criteria for access to confidential data.*<br><br>*The data underlying the results presented in the study are available from (include the name of the third party*	This work is based on publicly available papers and so is in the public domain.

and contact information or URL).<br>• This text is appropriate if the data are owned by a third party and authors do not have permission to share the data.<br><br>* typeset	
Additional data availability information:	

Manuscript

Click here to access/download;Manuscript;ACEs Systematic review.docx

A systematic review of non-clinician trauma-based interventions for school-age children and young people

Short title: Review of non-clinical trauma-based interventions for school children.

Flo Avery[1], Natasha Kennedy[1], Michaela James[1], Hope Jones[1], Rebekah Amos[2], Mark Bellis[3], Karen Hughes[2,4], Sinead Brophy[1*]

[1] National Centre for Population Health and Wellbeing, School of Medicine, Swansea University, Swansea, United Kingdom.

[2] School of Human Sciences, Bangor University, Wrexham, United Kingdom

[3] Faculty of Health, Liverpool John Moores University, Liverpool, United Kingdom

[4] Policy and International Health Directorate, World Health Organization Collaborating Centre on Investment for Health and Well-Being, Public Health Wales, Wrexham, United Kingdom

* Corresponding author

Email:s.brophy@swansea.ac.uk

Abstract

Exposure to adverse childhood experiences (ACEs) is increasingly recognised as a risk factor for health problems in later life. Awareness of ACEs and associated trauma is increasing in schools and educational settings, as well as demand for supportive services to deal with needs. However, there is a lack of clear evidence for effective interventions which can be delivered by non-clinicians (e.g., the school staff themselves). For this review, we conducted a search across five electronic databases for studies published since January 2013. These studies assessed the efficacy of non-clinician delivered interventions for supporting trauma recovery or improvements in mental health in school age youth (4-18 years) who have experienced ACEs. Out of the 4097 studies identified through the search, 326 were retrieved for full text screening and 25 were included in the final review. There was considerable heterogeneity in study design, outcome measures and the intervention being studied. The majority of studies were assessed to be of weak quality due to convenience sampling of participants and potential bias, indicating there is a lack of high quality research evidence to inform non-clinician delivered trauma-informed interventions for improving mental health outcomes in school-age young people. Cognitive Behavioural Therapy (CBT)-based approaches are tentatively suggested as a suitable target for future rigorous evaluations of interventions addressing ACE-related trauma recovery and mental health improvement in school-age young people.

Introduction

Adverse Childhood Experiences (ACEs) are potentially traumatic experiences such as child abuse or neglect, or household dysfunction (such as drug and alcohol abuse or domestic violence within the family home), occurring before the age of 18. Young people exposed to ACEs face an elevated risk of unhealthy behaviours and long term physical and mental health problems (1,2). This correlation is particularly pronounced among those who have experienced four or more ACEs (3). While not everyone who has ACEs necessarily experiences a traumatic event (4), the occurrence of multiple ACEs closely predicts the development of trauma symptoms (5).

Promoting ACE awareness and implementing trauma-informed services are increasingly recommended as vital supportive measures, both in healthcare (6) and educational (7) settings. In response, schools and policy-makers are advocating for and embedding trauma-informed approaches (8,9). Schools and educational settings are recognised as crucial settings for providing support to young people affected by potentially traumatic experiences (10).

Numerous reviews have examined the evidence for different systemic approaches and therapeutic interventions for supporting young people with trauma (11–13). Many of the interventions in these reviews are clinical in nature, delivered by psychologists, therapists, or medical and allied health professionals. However, with ACEs being relatively common, for example between 1%-38% (3) reported to experience 4 or more ACE, a more population approach is needed. Yet, there is a notable gap in evidence for interventions suitable for non-clinician delivery (14). This is a barrier for many schools with limited access to clinicians and without mental health professionals on staff. Consequently, teachers and teaching assistants are

often tasked with providing mental health support to young people (15,16). While Franklin et al. (17) have suggested that interventions delivered by teachers and non-clinicians can be beneficial for young people's mental health, they highlight the lack of empirical evidence regarding their effectiveness.

This review, therefore, aims to address the question of what evidence base exists for interventions supporting trauma, specifically those suitable for non-clinician delivery. The objective is to synthesise evidence for interventions appropriate for professionals such as teachers and teaching assistants, acknowledging their increasingly critical role in providing mental health support to young people affected by potentially traumatic experiences.

Methods

This systematic review was conducted in accordance with the Preferred Reporting Items for Systematic Reviews and Meta-Analysis (PRISMA) guidelines (18). The protocol for the review was registered in the PROSPERO International Prospective Register of Systematic Reviews in June 2023 (ID: CRD42023417286).

Review Design

We conducted a systematic review of quantitative and qualitative evidence to evaluate the effectiveness of trauma-informed interventions suitable for non-clinician delivery. The study

findings were summarised through a narrative synthesis. Heterogeneity in study designs and outcome measures precluded the ability to conduct a meta-analysis. One researcher (FA) extracted study data from full text papers, and this was reviewed by a second researcher (RA). Any inaccuracies were presented to and resolved by a third independent researcher

Search Strategy

Five electronic databases – Web of Science, Embase, Science Direct, Applied Social Sciences Index (ASSIA) and EBSCO (including CINAHL Plus with Full Text, MEDLINE, APA PsycArticles, APA PsycInfo, Teacher Reference Center, and Education Research Complete) – were searched to identify relevant studies published in English. The literature search was conducted in April 2023. Search terms included: Trauma* OR "Post-Traumatic Stress" OR PTSD and Intervention* OR Treatment* and children OR youth OR young OR adolescen* and education OR school OR teach* OR play. Detailed search terms can be viewed in Appendix 1.

Eligibility

Inclusion Criteria

Articles were included if they met the following criteria: (1) published in English in the last 10 years (e.g since January 2013); (2) reported on any supportive or therapeutic intervention with applicability to trauma recovery, or recovery from the impact of ACEs; (3) intervention was suitable for non-clinician delivery e.g. teacher, teaching assistant or similar; (4) participants were aged 4-18 years (school aged) with any experience of or exposure to ACEs; (5) intervention took place in a school, educational setting, community setting, residential or care

settings; and (6) included a validated self-reported mental health outcome relating to trauma or adversity such as post-traumatic stress disorder (PTSD) symptoms as an outcome measure. Both randomised and non-randomised studes were included. Language of publication was restricted owing to constraints of the research team's capacity. Restrictions were made by publication date to maximise the relevance of studies.

Exclusion Criteria

Articles were excluded if they met the following criteria: 1) All participants were over 18 or under 4 years; 2) interventions were assessed indirectly through a teacher or professional assessment of young people; 3) interventions were based on disasters such as earthquakes, floods or experiences of war; 4) interventions were delivered by clinicians; 5) interventions were behavioural rather than supportive, such as violence prevention or sex education programmes; 6) the research study had implemented a case study designs; and 7) the research had been conducted in a hospital or in-patient healthcare setting.

Study Selection

The search yielded 7147 results, which were imported into Covidence for title and abstract screening. After removing 3050 duplicates, the remaining titles and abstracts (4097) were screened against the selection criteria by the primary author, FA. Additionally, all titles and abstracts underwent independent screening by a second reviewer. Reviewers achieved a 96% agreement rate, and any disagreements were resolved through discussions among authors (FA, SB, NK, RA, HJ, MJ), resulting in a majority consensus reached by agreement between at least three authors. As a result, 3770 studies were excluded as irrelevant. FA retrieved and

independently screened all full-text articles (326), which were also independently screened by a second reviewer. Reviewers reached an 88% agreement rate, resolving disagreements through discussion amongst authors (FA, SB, NK, HJ, MJ), with a majority vote from at least three authors. Figure 1 shows details of the selection process, including reasons for excluding articles at different stages. Twenty-four articles met the inclusion criteria. Data extraction from these articles was undertaken by one reviewer (FA) and cross-checked by a second reviewer (RA or MJ). Data extracted included demographic data regarding the papers such as country of study, target population, year of publication, and descriptive information about the intervention such type of intervention, length of followup, measures of effect, barriers to implementation. The outcomes differed in each study and so a descriptive extraction of the data in each study was recorded. The study quality was assessed using validated tools (see below). Furthermore, reference lists from these twenty four studies were manually searched by FA, resulting in the identification of one additional paper. This was uploaded to Covidence and voted on by other authors for inclusion. The paper was voted eligible for inclusion and so a total of twenty-five articles met the inclusion criteria and are included in this review.

Quality Assessment

Effective Public Health Practice Project (EPHPP)'s Quality Assessment Tool for Quantitative Studies was used to assess the quality of quantitative research including randomised control trials (RCTs), non-randomised experimental studies and cohort design studies (19). This tool assesses potential selection bias, the appropriateness of study design, confounding, blinding, data collection tools, participant dropouts, intervention integrity and statistical analyses. Quality ratings are scored on a scale of 1-3, where 1 indicates strong quality, 2 indicates moderate quality, and 3 indicates weak quality. Overall ratings are classified as a) weak (when

2 or more components rated as weak), b) moderate (when only one component rated as weak), or c) strong.

Qualitative studies were assessed using the Critical Appraisal Skills Programme (CASP) Qualitative Studies Checklist (20). This checklist assesses three broad areas, such as a) the validity of the study, b) the study findings and c) the utility of the research locally. Within each of these broad domains are a set of questions designed to provide an overview of the study quality in each domain (20). Mixed methods research was assessed using both checklists. No studies were excluded based on the results of the quality assessment. Quality assessment was conducted by one reviewer (FA) and checked by a second reviewer (RA and MJ). Disagreements were resolved through discussion amongst authors.

Results

Overview of studies

Characteristics of the twenty five studies included in the current review are summarised in Table 1. Six studies were randomised control trials (21–26), fifteen used a cohort design (pre-test and post-test) (26–38), three used qualitative methods (39–41) and one used mixed methods (cohort design and qualitative methods) (42). The average sample size was 119 participants, ranging from 15 participants (37) to 565 participants (43). Of the included studies, 14 (56%) had a sample size above 50 (21,27,28,30,32,34–36,39,43–46). As anticipated, there was a high level of methodological diversity in terms of study design, populations (e.g. refugee, LGBTQ, youth offender, middle east, USA, Europe, Asia) and measures used, and therefore meta-analysis was not conducted.

The studies in this review either recruited the study population based on likely exposure to trauma, measured exposure to traumatic events, or measured PTSD symptoms (or a mix of these). Seven studies directly measured exposure to traumatic events (21,22,25,30,35,40,44,47). Thirteen studies directly measured PTSD symptoms (22,24,29,34,40,42), and of these, seven used PTSD score as selection criteria (29,30,33–35,37,43).

Ten studies assessed interventions which were delivered in the school setting (24,25,28–30,33,35,36,38,43). Three studies observed interventions delivered online (22,34,37). Eight studies looked at interventions delivered in a community setting (such as a church or charity site) (21,23,26,27,31,39,44,46), two were interventions delivered on university campuses (40,41), and one was an intervention delivered in a residential setting (32). One study evaluated the intervention across different settings, including a school and residential setting (42).

Quality of included studies

The quality assessment scores of included studies are shown in Tables 2-4. The methodological quality of included studies was weak overall. Using the EPHPP tool for quantitative studies, two studies were categorised as strong, seven studies as moderate, and the rest as weak. The majority of studies rated as weak relied on convenience samples of participants and did not adequately describe methodological information, such as potential confounding and blinding. Overall, qualitative studies were not considered strong, primarily because they lacked consistent discussions on how the relationship between researcher and participants was managed, including consideration of power dynamics and ethical issues.

The nine studies rated moderate or strong on quality included a range of interventions. Two studies looked at CBT-based groups in refugee populations (29,43). Other studies evaluated interventions focused on expressive writing (32), mindfulness (33), game-based mindfulness (37), trauma-focused CBT (delivered by lay counsellors) (44), a targeted group for sexual and gender minority adolescents (SGMA) (24), and a psychoeducational programme about posttraumatic growth (25).

CBT-based groups

Seven studies evaluated CBT-based groups, although four were of weak quality. Four studies investigated Teaching Recovery Techniques (TRT), a five week programme based on trauma-focused cognitive behavioural therapy (TF-CBT) designed for children in low resource settings, making it suitable for delivery by non-clinicians. The program was developed by the Children and War Foundation (48). El-Khani et al. (43) evaluated TRT in an RCT with Syrian refugee children aged 9-12 and their families in Lebanon, comparing it with a waitlist control group and a TRT + parenting (TRT+P) condition, which was rated as strong for quality. Data were collected pre-intervention (T1) and post-intervention at 2 weeks (T2) and 12 weeks (T3). All three conditions experienced a significant reduction in PTSD intrusion symptoms measured on the CRIES-13, with the greatest reduction in the TRT+P condition, showing the lowest levels at T3. Both the TRT and TRT+P condition also experienced reductions in depression. Furthermore, all three conditions experienced a significant reduction in anxiety, however the waitlist group’s scores increased by T3. It is suggested that a parenting skills component can enhance the positive effects of TRT. A pilot study by the same authors evaluating TRT in the same population in a Turkish setting was also included (29). This study was rated weak for

quality due to selection bias of participants and the absence of a control group. Similar findings were observed; a significant reduction was reported by participants in the Intrusion scores on the CRIES-13 at two weeks post-intervention, although other measures did not reach significance owing to low power (n=16).

Sarkadi et al. (42) evaluated TRT in an adolescent refugee population in Sweden and found significant reductions in PTSD (CRIES-8) and depression (MADRS-S) symptoms two weeks after the intervention. Qualitative data indicated that the programme had utility through provision of social support, normalisation, valuable tools, and manageability. However, there was no reported follow up beyond two weeks; a 3-6 month follow up was planned, but measures were not reported. Barron (26) found similar results in their RCT evaluating the impact of TRT for youth with PTSD in Brazilian favelas, showing a significant reduction in PTSD and depression symptoms, and a small effect size for posttraumatic growth compared with the control group. However, there was no follow up beyond two weeks post-intervention, providing no evidence of long term impact. TRT was the most extensively studied intervention and has the strongest evidence base for reducing PTSD symptoms, although two of these studies were rated as low quality (29,49), and only one followed up beyond two weeks post-intervention (43).

Bryant et al. (21) conducted a moderate-quality RCT evaluating a similar group-based intervention called Early Adolescent Skills for Emotions (EASE) for Syrian adolescents. The intervention spanned seven group sessions focusing on arousal reduction, behavioural activation, and problem management. Data was collected pre-intervention (T1) and post-intervention at 9 weeks (T2) and 3 months (T3). Significant reductions in the PSC-internalising scale were found compared to the control group, and this effect was sustained at T3, although

no significant difference were found in other variables. EASE was also evaluated by Akhtar et al. (46) in a pilot RCT, although the study was low powered and did not detect any significant changes.

Elswick et al. (30) evaluated the impact of a group after school club called the Trauma Healing Club, based on Cognitive Behavioral Interventions for Trauma in Schools (CBITS). CBITS is an evidence-based intervention deliverable by a clinician, whilst the Trauma Healing Club is suitable for non-clinicians. While significant improvement in PTSD symptoms was observed, it should be noted that this was a poor-quality study that did not report on withdrawals, the reliability and validity of measures, or potential confounders.

Li et al. (30) assessed the Power up Children's Psychological Immunity (PCPI) program, a modified group version of TF-CBT. This study was also of poor-quality and did not report on reliability and validity of measures, or potential confounders. Participants reported significantly reduced PTSD, depression, and anxiety following the intervention, but by the 3 month follow up, levels were similar to pre-intervention levels.

Overall CBT-based groups had the strongest evidence base of any intervention type, with three RCTs rated strong or moderate quality providing promising evidence. However it should be noted that the remainder of studies evaluating CBT-based groups were rated weak in quality and are therefore of limited utility.

Other CBT-based Approaches

Four studies explored CBT-based approaches in three distinct further contexts; one-to-one interventions (44,47), an online platform (34) and an art-based curriculum (38).

Murray (44) conducted an evaluation of TF-CBT for children affected by HIV/AIDS in Zambia. This was a modified lay counsellor-delivered version of the intervention, which is typically administered by clinicians. The sessions involved a combination of individual sessions with the child, caregiver(s) separately, and family sessions. Lay counsellors, lacking formal qualifications in counselling or mental health, typically provide psychological support through a community setting, often with a faith-based focus. This study received a moderate quality rating, revealing significant reductions in the severity of trauma symptoms and shame symptoms when comparing pre-intervention and two weeks post-interventionscores. However, there was no long term follow up. The authors did not provide a detailed description of how TF-CBT was adapted for non-clinician delivery, but it included cultural adaptations and simplified terminology. Close weekly monitoring was provided by the trainer and a supervisor to ensure fidelity.

Durmoney et al. (47) evaluated the impact of a peer delivered trauma-informed CBT skills curriculum among young men aged 17-24 years involved in the justice system. The intervention was delivered by ‘Paraprofessionals’ such as youth workers and volunteers in community settings, often within participants’ homes or on the street, to maximise engagement. This study was of weak quality, with a high likelihood of selection bias and unreliable or unverified data collection tools. Although there were indications of significant improvements in distress related to employment and education over time, emotion regulation did not show significant improvement.

Jaycox et al. (34) assessed an online stress and trauma curriculum called Life Improvement for Teens (LIFT), incorporating elements resembling TF-CBT and CBITS. The self-paced, internet-based curriculum was delivered online to high school students, who accessed the intervention during a weekly slot at the school site. LIFT comprised both a stress track and a trauma track, with students who reported potentially traumatic experiences being allocated to the trauma track. This study found significant improvements in PTSD symptoms and emotional problems, but observed no change in depression or anxiety. School functioning deteriorated over the course of the study. Overall, this study was rated as weak quality, as it did not specify the validity of data collection tools, and it did not discuss reasons for participant withdrawal.

Sitzer and Stockwell (36) also evaluated an art programme which incorporated aspects of CBT, which is discussed in the subsequent Art and community-based approaches section.

Mindfulness

Three studies focussed on evaluating a mindfulness intervention (22,33,37). Ito et al. (33) assessed the effects of short-term mindfulness-based group intervention delivered within a school setting for adolescents with trauma. Participants attended a single 60-minute group session and were subsequently encouraged by teachers to continue using mindfulness skills and cognitive defusion techniques. The study revealed significant improvements in mindful attention and awareness, along with reductions in depression, anxiety symptoms, and posttraumatic stress symptoms related to hyperarousal. However, these measures were recorded two weeks after the intervention, thus long term effects are unclear. This study was rated moderate quality. Schuurmans et al. (35) compared the impact of three meditation-based online games among youth in a residential care setting. Participants engaged in twelve 15-

minute sessions twice a week for 6 weeks. One game, called Muse, demonstrated improvements in post-traumatic symptoms, stress, and anxiety at the one month follow-up, while results for the other games were inconsistent. Although the study had a moderate overall quality rating, it featured a very small sample size, with n=15 at randomisation and n=9 at the one month follow up. Davis and Aylward (22) evaluated the impact of a trauma-informed mindful yoga intervention, which was delivered remotely to high school students. However, the study did not find significant differences in depression, anxiety, or resilience following the intervention.

Art and community-based approaches

Five studies evaluated interventions based on art, culture, or community, including two cohort design studies (27,38) and three qualitative studies (39–41). All studies received a weak quality rating. Sitzer and Stockwell (38 assessed a Wellness Programme delivered in schools, drawing on elements of art therapy along with aspects of CBT and Dialectical Behavioural Therapy (DBT). Results indicated significant increases in resilience and social and emotional functioning, particularly among male students. However, only unvalidated measures were used in this study. Barnett et al. (27) assessed the impact of residential culture camps on the wellbeing of Alaska Native youth aged 13 to 18 years. The results suggested a significant increase in positive affect, self esteem, and a sense of belonging. However, not all measures in this study were validated.

Harden et al. (40) examined the effects of a twice weekly after school programme involving media and theatre activities as youth-led empowerment strategies, conducted on a university

campus. The study identified positive themes related to empowerment, post-traumatic growth, and peace restoration among participants. Nevertheless, there were numerous methodological issues. The stated aim of the study was 'to contribute to a narrative that would reflect the TNT project's value', which is biased. There was no consideration of the relationship between researchers and participants, or ethical issues. These methodological weaknesses were common across all included qualitative studies.

Özden Bademci et al. (41) also evaluated a programme held on a university campus, featuring creative workshops and mentoring from volunteer university students for homeless boys aged 14-17 years. Qualitative themes indicated that participants perceived the campus as a safe, engaging environment, developed trusting relationships with mentors, and increased their capacity for emotional regulation. However, similar methodological critiques applied, including a lack of consideration for the potential ethical issues arising from the mentoring relationships, and insufficient detail provided concerning the interview protocol. McMahon & Pederson (39) assessed a community-based juvenile justice diversion program and identified positive themes related to self-efficacy, empathy, connection, and conflict resolution. However, the research protocol, including how themes were reached, was not described. Overall, the evidence for art and community-based approaches found to be very weak.

Other interventions

The remaining interventions, which did not fit into thematic categories, will be summarised individually. Greenbaum & Javdani (32)2 evaluated a therapeutic writing intervention called Writing and Reflecting on Identity To Empower Ourselves as Narrators (WRITE ON), delivered to juvenile justice-involved youth. The intervention consisted of 90 minute sessions

twice a week for six weeks. The study observed significant increases in positive mental health attributes and resilience throughout the programme and at a two week follow up. The quality was strong, although the long term impact remained unclear.

Goldbach et al. (24) conducted an RCT to assess a 10 week intervention called Proud & Empowered (P&E), which targeted SGMA and was conducted during the school day. Participation in the P&E intervention reduced minority stress and improved depression and suicidality measures, although there was no significant change in PTSD symptoms. Data was collected only one week after the intervention finished. This study received a moderate quality rating overall; validity and reliability of measures was not stated.

Taku et al. (25) examined a pychoeducational intervention about posttraumatic growth (PTG) in three conditions: a Control Group, Group 1 (intervention focused on stress-related reactions and PTG), and Group 2 (focused on negative changes and PTSD only). In Study 1, Group 1 and the Control Group showed higher PTG than Group 2 after two weeks, suggesting that exposure to negative information about PTSD may supress PTG. In Study 2, there were no differences in PTG perceptions between the groups. Findings were inconclusive for this moderate quality study.

Eruyar and Vostanis (31) evaluated the impact of group Theraplay, an attachment based intervention, with refugee children and their parents. After eight weekly sessions, a significant improvement was found in PTSD scores. However, only 59% of participants completed the programme, and reasons for withdrawals were not provided. Additionally, not all data collection tools were validated, although the PTSD measure was, which limited the quality.

This study was one of five studies, of varying quality, assessing interventions involving both children and caregivers, all of which suggested a positive impact (29,31,42–44).

Day et al. (28) assessed a trauma-informed intervention in a residential school, involving curriculum changes and access to relational interventions such as Theraplay to build attachment, self-esteem, and trust in others. Provision was made for two rooms to act as alternative environments for struggling students, providing access to problem solving, talk therapy, and use of sensorimotor activities. Significant differences were found in the pre and postest scores for posttraumatic symptoms. However, the study was weak in quality given the high chance of selection bias and a lack of validated measures. Furthermore, the intervention was broadly described without specific details.

Martin and Wood (36) evaluated a group drumming intervention delivered in a school and found significant improvements for boys in terms of higher mental wellbeing and lower post-traumatic stress symptoms, but not for girls. This study was of weak quality due to selection bias and a high unexplained drop out rate.

Discussion

A systematic review methodology was used to investigate the evidence base for interventions designed to support young people affected by trauma and are suitable for delivery by non-clinicians. This is the first systematic review to comphrensively appraise interventions in this domain specifically designed for non-clinician delivery. Overall, the evidence base for trauma recovery interventions was found to be weak. Only nine out of the twenty-five studies identified were rated as strong or moderate quality, and among these, only seven reported significant

improvements in mental health outcomes, including depression, anxiety, or PTSD (21,26,32,33,37,43,44). Among these seven, four adopted a CBT-based approach (21,26,43,44), while two followed a mindfulness approach (33,37). The most frequently studied CBT-based intervention was TRT (26,43,49). A number of studies evaluating TRT were excluded earlier in the review process because they were administered by a clinician. While TRT itself is suitable for non-clinician delivery, some excluded studies involved clinician delivery to enhance fidelity. CBT-based groups, particularly TRT, are tentatively suggested as the interventions in this domain with the strongest evidence base.

Several of the included studies trialled interventions with refugee populations (21,29,30,43,46,49,50). This area has a growing evidence base, considering that refugees are often supported in low-resource environments such as refugee camps, where accessing specialised support can be challenging even after achieving settled status. Notably, this review excluded studies specifically measuring war- or disaster-related trauma. As a result, the studies which have been included from this context are more generalisable to a wider range of young people affected by adversity.

Six studies evaluated interventions which involved caregivers in some or all of the sessions which formed the intervention (29,43,44,46,49,50). El-Khani et al. (43) observed that TRT achieved a greater positive impact on young people’s mental health and PTSD symptomology when a parenting component was included. This finding suggests a potential avenue for future research, given the intergenerational nature of ACEs and the recognised role of trusted relationships with close adults as a potential protective factor for young people exposed to ACEs (51).

Implications for research

In this domain, there is a notable scarcity of high quality studies, highlighting the need for further RCTs to assess the potential impact of interventions. CBT-based groups and mindfulness interventions appear to be promising targets for future research. Rather than relying solely on clinician-delivered designs to ensure fidelity of interventions intended for non-clinicians, more robust results can be derived from studies that implement non-clinician delivery, supplemented with measures such as observation or clinical supervision to maintain fidelity. Additionally, it is essential to incorporate longer-term follow up into study design. A majority of studies included in this review collected post-intervention data within a period of 2 weeks or less. To establish the robustness of intervention evidence, longer-term follow up assessments are imperative.

In situations where conducting RCTs may not be feasible, efforts should be directed towards ensuring the reliability and validity of data collection tools. Furthermore, researchers should diligently record and follow up on withdrawals from the study. This practice is both methodologically and ethically important, as traumatised youth may disengage from therapeutic interventions if they perceive them as harmful or unsupportive. Exploring this perspective can provide valuable insights into improving intervention strategies.

Implications for educational settings

There is not a strong, definitive recommendation for schools seeking to adopt a research-informed approach within their educational setting to support young people who have experienced trauma and adversity. However, based on available evidence, the approaches

which are most likely to be effective at supporting young people with trauma recovery are CBT-based groups or mindfulness approaches. Additionally, interventions involving caregivers may also have value in this context. This aspect might be particularly pertinent for primary schools, which serve pupils aged under 11 years in the United Kingdom, and often maintain closer partnerships with parents and caregivers.

Review limitations

The overall quality of this systematic review was assessed using AMSTAR 2 (52). The review meets or partially meets all checklist items on AMSTAR 2. Although the time frame for follow up was not stated in the inclusion crtieria, it has been reported for all strong and moderate quality studies. The review did not examine study registries and did not seek to consult experts in the field and sources of funding for the studies included in the review have not been reported. Given the high level of anticipated and actual heterogeneity of studies in this area, a meta-analysis was not planned or conducted, which restricts the scope of the conclusions that can be drawn. Finally, the work only examined studies from the past ten years, only selected those published in English, and may have missed those published in non-academic literature. The secondary screen of the references of papers may have help to identify some of the articles missing due to the search criteria, but future work would be needed to idenifty interventnions published in grey literature or published in lanuages other than English.

Conclusions

Our review of 25 studies reveals inconsistent evidence regarding non-clinician delivered trauma informed interventions for enhancing mental health outcomes in school-age

individuals. There are a number of strategies with evidence that would suggest more development and rigorous evaluation would be of benefit. That is especially including an economics or cost effectiveness evaluation of those delivered online or further evaluation of the CBT-based approaches. As schools and educational institutions are increasingly expected to assume a greater role in supporting mental health of traumatised youth, the development of this evidence base, is of crucial importance.

Avaliablity of materials:

Data collection templates, the Covidence search results and any other materials are available upon request to s.brophy@swansea.ac.uk.

Funding

This research was supported by the National Centre for Population Health and Wellbeing Research. Funding was provided by Public Health Wales and the Economic and Social Research Council (ESRC) through their support of a PhD studentship.

Author Contributions

Flo Avery led the review, was responsible for managing the project, analysing data and preparing an original draft.

Michaela James contributed to abstract and full text screening, quality assessment and editing.

Rebekah Amos contributed to abstract and full text screening, and quality assessment, editing and writing.

Natasha Kennedy contributed to abstract and full text screening, and editing.

Hope Jones contributed to abstract and full text screening, and editing.

Sinead Brophy, Karen Hughes and Mark Bellis provided supervision and advice for the systematic review and methodology, and reviewed the final report.

Competing interests: None to declare

References

1. Anda RF, Felitti VJ, Bremner JD, Walker JD, Whitfield C, Perry BD, et al. The enduring effects of abuse and related adverse experiences in childhood. European Archives of Psychiatry and Clinical Neuroscience 2005 256:3 [Internet]. 2005 Nov 29 [cited 2023 Jun 16];256(3):174–86. Available from: https://link.springer.com/article/10.1007/s00406-005-0624-4
2. Bellis MA, Hughes K, Leckenby N, Hardcastle KA, Perkins C, Lowey H. Measuring mortality and the burden of adult disease associated with adverse childhood experiences in England: A national survey. Journal of Public Health (United Kingdom). 2015 Sep 1;37(3):445–54.
3. Bellis MA, Ashton K, Hughes K, Ford KJ, Bishop J, Paranjothy S. Adverse childhood experiences and their impact on health-harming behaviours in the Welsh adult population. Public Health Wales NHS Trust; 2016.
4. Finkelhor D. Screening for adverse childhood experiences (ACEs): Cautions and suggestions. Child Abuse Negl. 2018 Nov 1;85:174–9.
5. Finkelhor D, Ormrod RK, Turner HA. Poly-victimization: A neglected component in child victimization. Child Abuse Negl. 2007 Jan 1;31(1):7–26.

6. Hughes K, Bellis MA, Hardcastle KA, Sethi D, Butchart A, Mikton C, et al. The effect of multiple adverse childhood experiences on health: a systematic review and meta-analysis. Lancet Public Health. 2017 Aug 1;2(8):e356–66.
7. Wassink-De Stigter R, Kooijmans · R, Asselman · M W, Offerman · E C P, Nelen · W, Helmond · P. Facilitators and Barriers in the Implementation of Trauma-Informed Approaches in Schools: A Scoping Review. School Ment Health [Internet]. 2022 [cited 2023 Jun 16];14:470–84. Available from: https://doi.org/10.1007/s12310-021-09496-w
8. Temkin D, Harper K, Stratford B, Sacks V, Rodriguez Y, Bartlett JD. Moving Policy Toward a Whole School, Whole Community, Whole Child Approach to Support Children Who Have Experienced Trauma. Journal of School Health [Internet]. 2020 Dec 1 [cited 2023 Jun 21];90(12):940–7. Available from: https://onlinelibrary.wiley.com/doi/full/10.1111/josh.12957
9. Berger E, Martin K. Embedding trauma-informed practice within the education sector. J Community Appl Soc Psychol [Internet]. 2021 Mar 1 [cited 2023 Jun 21];31(2):223–7. Available from: https://onlinelibrary.wiley.com/doi/full/10.1002/casp.2494
10. Hoover S, Bostic J. Schools as a vital component of the child and adolescent mental health system. Psychiatric Services [Internet]. 2021 Jan 1 [cited 2023 Jun 21];72(1):37–48. Available from: https://ps.psychiatryonline.org/doi/10.1176/appi.ps.201900575
11. Black PJ, Woodworth M, Tremblay M, Carpenter T. A review of trauma-informed treatment for adolescents. Canadian Psychology. 2012;53(3):192–203.
12. Lange BCL, Nelson A, Lang JM, Stirman SW. Adaptations of evidence-based trauma-focused interventions for children and adolescents: a systematic review. Implementation Science Communications 2022 3:1 [Internet]. 2022 Oct 8 [cited 2023 Feb 21];3(1):1–21. Available from:

https://implementationsciencecomms.biomedcentral.com/articles/10.1186/s43058-022-00348-5

13. Marie-Mitchell A, Kostolansky R. A Systematic Review of Trials to Improve Child Outcomes Associated With Adverse Childhood Experiences. Am J Prev Med. 2019 May 1;56(5):756–64.

14. Stratford B, Cook E, Hanneke R, Katz E, Seok D, Steed H, et al. A Scoping Review of School-Based Efforts to Support Students Who Have Experienced Trauma. School Ment Health [Internet]. 2020 Sep 1 [cited 2023 Jun 21];12(3):442–77. Available from: https://link.springer.com/article/10.1007/s12310-020-09368-9

15. Luthar SS, Mendes SH. Trauma-informed schools: Supporting educators as they support the children. https://doi.org/101080/2168360320201721385 [Internet]. 2020 Apr 2 [cited 2023 Jun 21];8(2):147–57. Available from: https://www.tandfonline.com/doi/abs/10.1080/21683603.2020.1721385

16. Ekornes S. Teacher Perspectives on Their Role and the Challenges of Inter-professional Collaboration in Mental Health Promotion. School Ment Health [Internet]. 2015 Sep 10 [cited 2023 Jun 21];7(3):193–211. Available from: https://link.springer.com/article/10.1007/s12310-015-9147-y

17. Franklin CGS, Kim JS, Ryan TN, Kelly MS, Montgomery KL. Teacher involvement in school mental health interventions: A systematic review. Child Youth Serv Rev. 2012 May 1;34(5):973–82.

18. Moher D, Liberati A, Tetzlaff J, Altman DG, Altman D, Antes G, et al. Preferred Reporting Items for Systematic Reviews and Meta-Analyses: The PRISMA Statement. PLoS Med [Internet]. 2009 Jul 1 [cited 2023 Jun 21];6(7):e1000097. Available from: https://journals.plos.org/plosmedicine/article?id=10.1371/journal.pmed.1000097

19. Evans, N., Lasen, M., & Tsey, K. (2015). Appendix A: Effective public health practice project (EPHPP) quality assessment tool for quantitative studies. *A systematic review of rural development research SpringerBriefs in Public Health*, 45-55.

20. *Critical Appraisal Skills Programme (2022). CASP) Checklist. [online] Available at:* CASP Checklists - Critical Appraisal Skills Programme (casp-uk.net). *Accessed: 24/09/2023*

21. Bryant RA, Malik A, Aqel IS, Ghatasheh M, Habashneh R, Dawson KS, et al. Effectiveness of a brief group behavioural intervention on psychological distress in young adolescent Syrian refugees: A randomised controlled trial. PLoS Med. 2022 Aug 1;19(8).

22. Davis L, Aylward A. Mitigating Rural Adolescent Trauma: Remote Delivery of a Trauma-Informed Yoga Intervention During COVID-19. J Child Adolesc Trauma. 2022;

23. Dumornay NM, Finegold KE, Chablani A, Elkins L, Krouch S, Baldwin M, et al. Improved emotion regulation following a trauma-informed CBT-based intervention associates with reduced risk for recidivism in justice-involved emerging adults. Front Psychiatry. 2022 Oct 5;13:951429.

24. Goldbach JT, Rhoades H, Mamey MR, Senese J, Karys P, Marsiglia FF. Reducing behavioral health symptoms by addressing minority stressors in LGBTQ adolescents: a randomized controlled trial of Proud & Empowered. BMC Public Health. 2021 Dec 1;21(1).

25. Taku K, Cann A, Tedeschi RG, Calhoun LG. Psychoeducational Intervention Program about Posttraumatic Growth for Japanese High School Students. J Loss Trauma. 2017 May 19;22(4):271–82.

26. Barron I, Freitas F, Bosch CA. Pilot Randomized Control Trial: Efficacy of a Group-Based Psychosocial Program for Youth with PTSD in the Brazilian Favelas. J Child Adolesc Trauma [Internet]. 2021 Sep 1 [cited 2023 Jul 20];14(3):335–45. Available from: https://link.springer.com/article/10.1007/s40653-020-00328-8
27. Barnett JD, Schmidt TC, Trainor B, Wexler L. A Pilot Evaluation of Culture Camps to Increase Alaska Native Youth Wellness. Health Promot Pract. 2020 May 1;21(3):363–71.
28. Day AG, Somers CL, Baroni BA, West SD, Sanders L, Peterson CD. Evaluation of a Trauma-Informed School Intervention with Girls in a Residential Facility School: Student Perceptions of School Environment. In: Journal of Aggression, Maltreatment and Trauma. Routledge; 2015. p. 1086–105.
29. El-Khani A, Cartwright K, Ang C, Henshaw E, Tanveer M, Calam R. Testing the feasibility of delivering and evaluating a child mental health recovery program enhanced with additional parenting sessions for families displaced by the Syrian conflict: A pilot study. Peace and Conflict. 2018 May 1;24(2):188–200.
30. Elswick S, Washington G, Mangrum-Apple H, Peterson C, Barnes E, Pirkey P, et al. Trauma Healing Club: Utilizing Culturally Responsive Processes in the Implementation of an After-School Group Intervention to Address Trauma Among African Refugees. J Child Adolesc Trauma. 2022 Mar 1;15(1):155–66.
31. Eruyar S, Vostanis P. Feasibility of group theraplay with refugee children in Turkey. Couns Psychother Res. 2020 Dec 1;20(4):626–37.
32. Greenbaum CA, Javdani S. Expressive writing intervention promotes resilience among juvenile justice-involved youth. Child Youth Serv Rev. 2017 Feb 1;73:220–9.

33. Ito D, Kubo Y, Takii A, Watanabe A, Ohtani T, Koseki S. The effects of short-term mindfulness-based group intervention utilising a school setting for Japanese adolescents with trauma. J Psychol Couns Sch. 2021 Dec 17;31(2):221–6.

34. Jaycox LH, Ayer L, Vona P, Hehman C, Stein BD, Mahmud A, et al. Development and preliminary evaluation of a self-guided, internet-based tool for coping with stress and trauma: Life improvement for teens (LIFT). Psychol Serv. 2019 Feb 1;16(1):85–94.

35. Li J, Li J, Zhang W, Wang G, Qu Z. Effectiveness of a school-based, lay counselor-delivered cognitive behavioral therapy for Chinese children with posttraumatic stress symptoms: a randomized controlled trial. Lancet Reg Health West Pac. 2023 Apr 1;33.

36. Martin KE, Wood LJ. Drumming to a New Beat: A Group Therapeutic Drumming and Talking Intervention to Improve Mental Health and Behaviour of Disadvantaged Adolescent Boys. Children Australia. 2017 Dec 1;42(4):268–76.

37. Schuurmans AAT, Nijhof KS, Scholte R, Popma A, Otten R. A novel approach to improve stress regulation among traumatized youth in residential care: Feasibility study testing three game-based meditation interventions. Early Interv Psychiatry. 2020 Aug 1;14(4):476–85.

38. Sitzer DL, Stockwell AB. The art of wellness: A 14-week art therapy program for at-risk youth. Arts in Psychotherapy. 2015 Sep 1;45:69–81.

39. McMahon SM, Pederson S. "Love and compassion not found Elsewhere": A Photovoice exploration of restorative justice and nonviolent communication in a community-based juvenile justice diversion program. Child Youth Serv Rev. 2020 Oct 1;117.

40. Harden T, Kenemore T, Mann K, Edwards M, List C, Martinson KJ. The Truth N' Trauma Project: Addressing Community Violence Through a Youth-Led, Trauma-

Informed and Restorative Framework. Child and Adolescent Social Work Journal. 2015 Feb 1;32(1):65–79.

41. Özden Bademci H, Figen Karadayi E, de Zulueta F. Attachment intervention through peer-based interaction: Working with Istanbul's street boys in a university setting. Child Youth Serv Rev. 2015;49:20–31.

42. Sarkadi A, Ådahl K, Stenvall E, Ssegonja R, Batti H, Gavra P, et al. Teaching Recovery Techniques: evaluation of a group intervention for unaccompanied refugee minors with symptoms of PTSD in Sweden. Eur Child Adolesc Psychiatry. 2018 Apr 1;27(4):467–79.

43. El-Khani A, Cartwright K, Maalouf W, Haar K, Zehra N, Çokamay-Yılmaz G, et al. Enhancing teaching recovery techniques (Trt) with parenting skills: Rct of trt + parenting with trauma-affected syrian refugees in lebanon utilising remote training with implications for insecure contexts and covid-19. Int J Environ Res Public Health. 2021 Aug 2;18(16).

44. Murray LK, Familiar I, Skavenski S, Jere E, Cohen J, Imasiku M, et al. An evaluation of trauma focused cognitive behavioral therapy for children in Zambia. Child Abuse Negl. 2013 Dec;37(12):1175–85.

45. Dumornay NM, Finegold KE, Chablani A, Elkins L, Krouch S, Baldwin M, et al. Improved emotion regulation following a trauma-informed CBT-based intervention associates with reduced risk for recidivism in justice-involved emerging adults. Front Psychiatry. 2022 Oct 5;13:951429.

46. Akhtar A, Malik A, Ghatasheh M, Aqel IS, Habashneh R, Dawson KS, et al. Feasibility trial of a brief scalable psychological intervention for Syrian refugee adolescents in Jordan. https://doi.org/101080/2000819820211901408 [Internet]. 2021

[cited 2023 Jul 25];12(1). Available from: https://www.tandfonline.com/doi/abs/10.1080/20008198.2021.1901408

47. Dumornay N, Finegold KE, Chablani A, Elkins L, Krouch S, Baldwin M, et al. Improved emotion regulation following a trauma-informed CBT-based intervention associates with reduced risk for recidivism in justice-involved emerging adults. Front Psychiatry. 2022;13.

48. Yule W, Dyregrov A, Raundalen M, Smith P. Children and war: The work of the Children and War Foundation. Eur J Psychotraumatol [Internet]. 2013 [cited 2023 Jul 25];4(SUPPL.). Available from: https://www.tandfonline.com/doi/abs/10.3402/ejpt.v4i0.18424

49. Sarkadi A, Ådahl K, Stenvall E, Ssegonja R, Batti H, Gavra P, et al. Teaching Recovery Techniques: evaluation of a group intervention for unaccompanied refugee minors with symptoms of PTSD in Sweden. Eur Child Adolesc Psychiatry [Internet]. 2018 Apr 1 [cited 2022 Nov 28];27(4):467–79. Available from: https://link.springer.com/article/10.1007/s00787-017-1093-9

50. Eruyar S, Vostanis P. Feasibility of group theraplay with refugee children in Turkey. Couns Psychother Res [Internet]. 2020 Dec 1 [cited 2023 Feb 21];20(4):626–37. Available from: https://onlinelibrary.wiley.com/doi/full/10.1002/capr.12354

51. Crouch E, Radcliff E, Strompolis M, Srivastav A. Safe, Stable, and Nurtured: Protective Factors against Poor Physical and Mental Health Outcomes Following Exposure to Adverse Childhood Experiences (ACEs). J Child Adolesc Trauma [Internet]. 2019 Jun 15 [cited 2023 Jul 25];12(2):165–73. Available from: https://link.springer.com/article/10.1007/s40653-018-0217-9

52. Shea BJ, Reeves BC, Wells G, Thuku M, Hamel C, Moran J, et al. AMSTAR 2: a critical appraisal tool for systematic reviews that include randomised or non-

randomised studies of healthcare interventions, or both. BMJ [Internet]. 2017 Sep 21 [cited 2023 Jul 21];358:4008. Available from: https://www.bmj.com/content/358/bmj.j4008

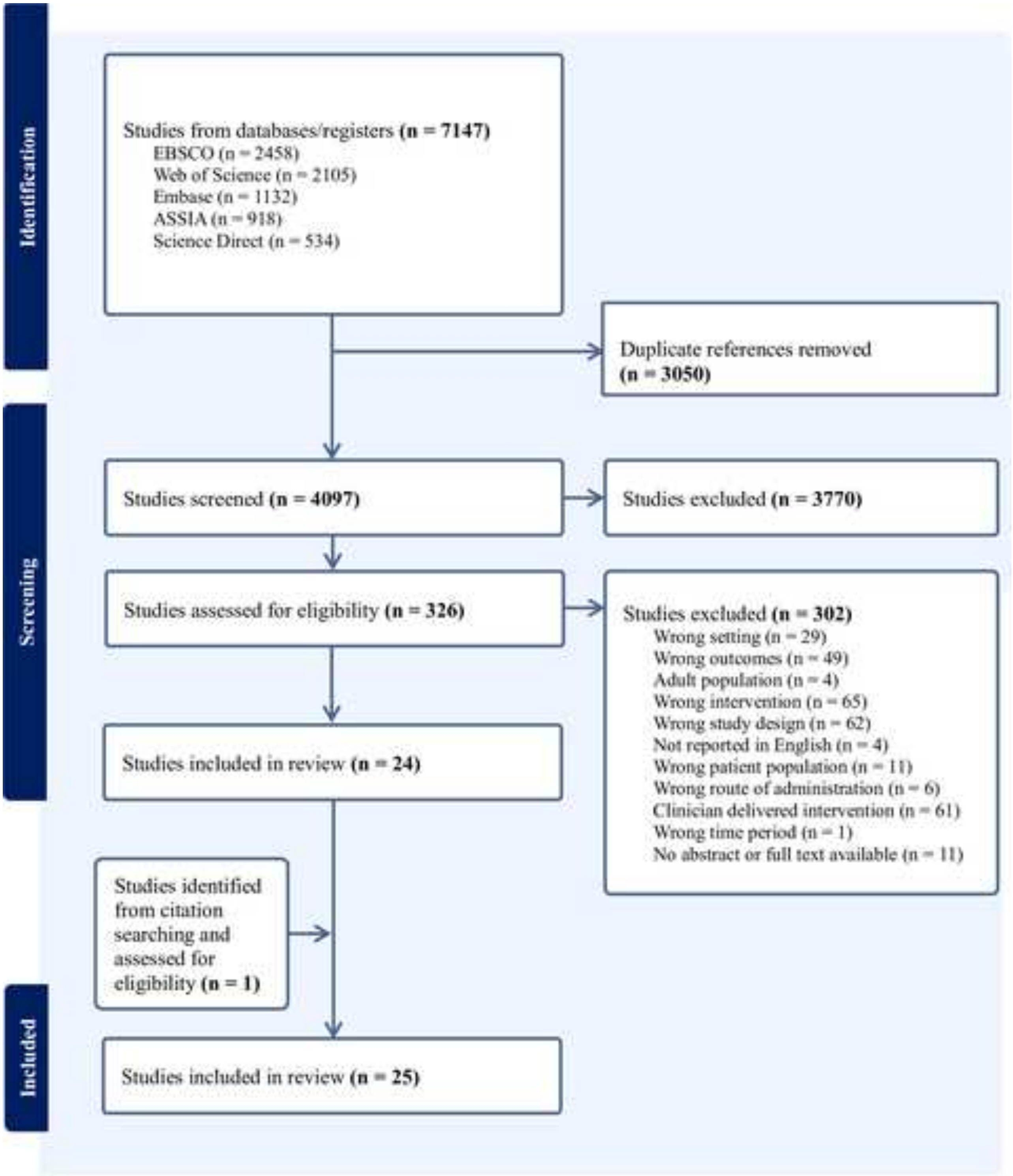

Fig 1. PRISMA diagram

Click here to access/download
Supporting Information
PRISMA_2020_checklist.docx
