# Supplementary material for: A systematic review of non-clinician trauma-based interventions for school-age youth
Source: PLoS One. 2024 Sep 6;19(9):e0293248. doi: 10.1371/journal.pone.0293248 (PMC11379276; doi:10.1371/journal.pone.0293248)
Supplement: Attachment — Submitted filename: Response to Reviewers Word.docx [file pone.0293248.s007.docx]

| Reviewer | Comment | Response | Original text | Altered text | Page | Line |
| --- | --- | --- | --- | --- | --- | --- |
| 1 | Suggested Title - Non-clinician trauma-based interventions for school-age children and young people: A systematic review | Thank you for your suggestion. The authors have discussed this change and we would like to use 'A systematic review of non-clinician trauma-based interventions for school-age youth' |  |  | 1 | 1 |
| 2 | To reduce confusion, I suggest that the title reads “… intervention for school-aged youth.” As this is clearly stated as such within the paper. I would also suggest that the authors choose how they will describe their population of interest and be consistent. Should they choose to use school-aged youth for instance, that should be throughout the whole paper. | Thank you very much for this feedback, we agree that using 'youth' throughout, where sentence structure permits, is more concise and clear. We have changed the terminology to 'school-age youth' throughout the paper | school-age children and young people | **school-age youth** | 1 | 2 |
| 2 | Line 22, where is this happening? (Abstract) | Thank you for this feedback. A number of studies around the world have found a link between ACEs and long term health, to clarify that this is not a geographically limited phenomena we have added word 'globally'. | Exposure to adverse childhood experiences (ACEs) is increasingly recognised as a risk factor for health problems in later life | Exposure to adverse childhood experiences (ACEs) is recognised **globally** as a risk factor for health problems in later life | 2 | 24 |
| 1 | Abstract: Add systematic review, it has to be clear which review you are using | Thank you for this comment. The abstract has been updated accordingly. | For this review | **Thus, we undertook a systematic review to answer the question:** | 2 | 29 |
| 2 | (Abstract)     What was the review question? | Thank you for highlighting this ambiguity. This has been reworded and amended in the abstract and at the end of the introduction to make it clearer. | These studies assessed the efficacy of non-clinician delivered interventions for supporting trauma recovery or improvements in mental health in school-age youth (4-18 years) who have experienced ACEs. ' | **Thus, we undertook a systematic review to answer the question: What evidence exists for the efficacy of non-clinician delivered interventions for supporting trauma recovery or improvements in mental health in school-age youth (4-18 years) who have experienced ACEs?'** | 2 | 29 |
| 2 | (Abstract)      Which search engines did you use? | Thank you for this suggestion. This information is provided in the search strategy on line 183. We have decided to not include this information in the abstract for conciseness. t is standard for published systematic reviews to not include this in the abstract. | No change | No change | 2 |  |
| 2 | (Abstract)       Which keywords or phrases did the authors use to search for data? | Thank you for this suggestion. This information is provided in the search strategy on line 192. We have decided to not include this information in the abstract for conciseness. It is standard for published systematic reviews to not include this in the abstract. | No change | No change | 2 |  |
| 2 | (Abstract)      Some of your leading selection criteria guides? | Thank you very much for this suggestion, this information has been added to the abstract. | We conducted a search across five electronic databases for studies published since January 2013 | We conducted a search across five electronic databases for studies published since January 2013 **that reported on interventions suitable for non-clinician delivery, were published in English in the last 10 years, and involved participants aged 4-18 years (school-age) that had exposure to ACEs.** | 2 | 32 |
| 2 | (Abstract)    Which appraisal tool did you use? | Thank you for this suggestion. It will not be possible to name the quality assessment tools in the abstract as PLOS One referencing guidance does not allow references in the abstract. We have added details of the assessment tools into the abstract. The names of the tools are detailed on line 168. |  | Selected studies were quality assessed using validated assessment tools. | 2 | 37 |
| 2 | (Abstract)    How did you analyze your data? | Thank you for this feedback. The analysis stage involves data extraction and quality assessment of articles selected to feature in the review. For clarity, this section of the abstract has been reworded. |  | Data were extracted from included articles for analysis and selected studies were quality assessed using validated assessment tools. | 2 | 36 |
| 2 | (Abstract)     Line 32 and 33, what does “weak quality, and high quality” imply? | Thank you for this feedback. The criteria for studies to receive a score of 'weak' has been briefly detailed e.g. 'due to convenience sampling of participants and potential bias'. Given other edits, it will not be possible to elaborate further as there is a 300 word limit for the abstract. Full details of how the quality assessment tools were used can be found in Tables 2-4. | No change | No change | 2 |  |
| 2 | (Abstract)  What are the findings of your review? From line 31-35 read as limitations of this review, line 35- 37 read as a recommendation. | Thank you for highlighting this. The absence of high quality evidence was a main finding. This section of the abstract has been reworded for clarity. | The majority of studies were assessed to be of weak quality due to convenience sampling of participants and potential bias | **A key finding was that** the majority of studies were assessed to be of weak quality due to convenience sampling of participants and potential bias | 2 | 40 |
| 2 | Introduction. May the authors kindly reference statement 46-48. | Thank you for this feedback, a reference for this statement has been added. | Adverse Childhood Experiences (ACEs) are potentially traumatic experiences such as child abuse or neglect, or household dysfunction (such as drug and alcohol abuse or domestic violence within the family home), occurring before the age of 18. Young people exposed to ACEs face an elevated risk of unhealthy behaviours and long term physical and mental health problems (1, 2) | Adverse Childhood Experiences (ACEs) are potentially traumatic experiences such as child abuse or neglect, or household dysfunction (such as drug and alcohol abuse or domestic violence within the family home), occurring before the age of 18 **[1].** Young people exposed to ACEs face an elevated risk of unhealthy behaviours and long term physical and mental health problems **[2]** | 3 | 105 |
| 2 | Line 52, which ACEs result in the development of trauma when experienced concurrently? | Thank you for this query. Any combination of ACEs makes the development of adverse mental health symptoms more likely, this is a finding which is well established in the literature. ACEs literature tends to consider the total ACEs 'score' as a variable, rather than distinguishing between different types of ACEs and specific risk profiles for each. | No change | No change | 3 | 106 |
| 2 | Line 64, where was this? | Thank you for this comment, this section of the paper has been reworded to provide clarity. | However, with ACEs being relatively common, for example between 1%-38% (3) reported to experience 4 or more ACE, a more population approach is needed. | **However, ACEs are common with around half of adults in general populations having suffered at least one ACE [14]. This is a significant proportion of the population, suggesting a broad approach to offering interventions for ACEs may be appropriate, such as offering through schools or other public services.** | 3 | 120 |
| 2 | Line 66, “ a more population approach is needed” what does that mean? Could this be rephased for clarity? | Thank you for this feedback. See above, this section has been reworded. |  |  | 3 | 121 |
| 2 | In the context which the authours are basing the review, what’s the status on ACE related trauma and counselling (both formal and informal)? | Thank you for this query. Formal counselling is beyond the scope of this review as it is delivered by a clinician (e.g. a counsellor or psychologist). We have added more detail on line 99 to clarify that counselllors are included within this category. Informal counselling is considered within this review. For example, the included Murray (2013) paper considers lay counselling delivered by non-clinicians. | This is a barrier for many schools, with limited access to clinicians and without mental health professionals on staff | This is a barrier for many schools, **which have limited access to clinicians such as counsellors and psychologists, and do not have mental health professionals on staff**. | 4 | 141 |
| 2 | Line 74, this research question tells us that the authors aim to look for the tools and their effectiveness. However, your abstracts only alludes to assessing the effectiveness hence one of my comments was asking about the available tools. It is important for the authors to be clearer with what they aim to do in the review and eliminate ambiguity. | Thank you for this comment. The review question has been reworded to eliminate ambiguity. | This review, therefore, aims to address the question of what evidence base exists for interventions supporting trauma, specifically those suitable for non-clinician delivery. The objective is to synthesise evidence for interventions appropriate for professionals such as teachers and teaching assistants, acknowledging their increasingly critical role in providing mental health support to young people affected by potentially traumatic experiences. | **The review question was: what evidence exists for the efficacy of non-clinician delivered interventions for supporting trauma recovery or improvements in mental health in school-age youth (4-18 years) who have experienced ACEs? The objective is to synthesise evidence from a systematic literature review process for interventions which are appropriate for professionals such as teachers and teaching assistants.** | 4 | 151 |
| 2 | -       Line 106 adolescent is incomplete. | Thank you for this feedback. We used the term adolescen* in the search as reported in the paper, this is deliberate to allow for adolescent and adolescence to be included. | No change | No change | 6 | 207 |
| 2 | -       Was this a world wide review? | Thank you for your feedback, yes this was a worldwide review. We have added further detail to inclusion criteria (5) to clarify this. | (5) intervention took place in a school, educational setting, community setting, residential or care settings; | (5) intervention took place in a school, educational setting, community setting, residential or care settings **in any country;** | 6 | 220 |
| 1 | Line 121 check spelling, is studes or studies? | Thank you for this comment. This error has now been corrected. | studes | **studies** | 6 | 222 |
| 2 | I think the last line 122-123 should be removed because that is already obvious from the 10 years section criteria mentioned. | Thank you for this comment. Although the 10 year limit has already been mentioned, this line provides a rationale for restricting by publication date and therefore we would like to keep it. | No change | No change | 6 | 224 |
| 2 | line 122-123 does bring up the question “ why 10 years?” which I would understand because interventions implemented for a longer period can easily show effectiveness as they are assessed overtime and may possibly be applied on different demographics. | Thank you for this query. There is no limit in the selection criteria on how long the intervention runs for. The 10 year publication limit only applies to when studies were published and was included in the selection criteria to maximise relevance of studies. Long term interventions or longitudinal studies are not excluded on this criteria (although, because these type of interventions tend to run for a matter of weeks not years, none were found) | No change | No change | 6 | 224 |
| 2 | -       In terms of your inclusion and exclusion criteria, the duration of intervention implementation; doesn’t that count? | Thank you for your comment. There were no exclusion criteria regarding the duration of intervention implementation, hence this has not been mentioned. | No change | No change | 6 |  |
| 2 | o   Studies screened, what type of screening was that? | Thank you very much for this question. The methods section details how many papers were screened by title and abstract, and how many were subsequently screened as full-text articles. This is the standard and widely accepted procedure for systematic literature reviews. | No change | No change | 7 |  |
| 1 | Line 183 You nee to clarify your selection process and literature search | Thank you for providing this feedback. The selection process and literature search is detailed under Study Selection. For additional clarity, more information has been added to this section. | After removing 3050 duplicates, the remaining titles and abstracts (4097) were screened against the selection criteria by the primary author, FA. | After removing 3050 duplicates, the remaining titles and abstracts (4097) were screened against the selection criteria by the primary author, FA, **and removed if they did not meet the selection criteria. If it was unclear from the title and abstract whether all the selection criteria were met, the article was allocated to full-text screening** | 7 | 248 |
| 2 | §  Studies excluded (n=3770), on what bases were they excluded? | Thank you very much for this comment. We did not record the reasons for removing articles at the title and abstract screening stage. Broadly they were removed if it was clear from the title and abstract that they did not meet the selection criteria. If there was any doubt about whether the exclusion criteria was met, the article was advanced to full text screening to make a more informed decision about exclusion. We have added further details of this process. | After removing 3050 duplicates, the remaining titles and abstracts (4097) were screened against the selection criteria by the primary author, FA. | After removing 3050 duplicates, the remaining titles and abstracts (4097) were screened against the selection criteria by the primary author, FA, **and removed if they did not meet the selection criteria. If it was unclear from the title and abstract whether all the selection criteria were met, the article was allocated to full-text screening.** | 7 | 248 |
| 2 | §  Kindly show when title, abstract and full screening was done and categorize reasons for exclusion accordingly (as stated in line 147) | Thank you for this feedback. The reasons for exclusion at the full text stage are provided in the PRISMA diagram. I have added the dates for when the title and abstract screening and the full text screening were completed. | As a result, 3770 studies were excluded as irrelevant. FA retrieved and independently screened all full-text articles (326), which were also independently screened by a second reviewer. Reviewers reached an 88% agreement rate, resolving disagreements through discussion amongst authors (FA, SB, NK, HJ, MJ), with a majority vote from at least three authors. Fig 1 shows details of the selection process, including reasons for excluding articles at different stages. | As a result, 3770 studies were excluded as irrelevant. **Title and abstract screening was completed on 23 June 2023.** FA retrieved and independently screened all full-text articles (326), which were also independently screened by a second reviewer. Reviewers reached an 88% agreement rate, resolving disagreements through discussion amongst authors (FA, SB, NK, HJ, MJ), with a majority vote from at least three authors. Fig 1 shows details of the selection process, including reasons for excluding articles at different stages. **The full text screening process was completed on 16 July 2023.** | 7 | 255 |
| 2 | Method of analysis is missing. | Thank you for this feedback. The analysis stage involves data extraction and quality assessment of articles selected to feature in the review. For clarity, further details have been provided under study selection. | . | **Analysis was carried out by assessing the quality of each study using validated tools, and synthesising the findings of the selected studies.** | 8 | 270 |
| 2 | o   May the authors please include that they also searched for studies via citation under study selection. | Thank you for this feedback. Citation searching is mentioned at the end of the study selection section. This section has been edited for clarity | Furthermore, reference lists from these twenty four studies were manually searched by FA, resulting in the identification of one additional paper. | Furthermore, reference lists **and citations** from these twenty four studies were manually searched by FA, resulting in the identification of one additional paper. | 8 | 272 |
| 2 | -       The results and discussion section need to be reworked overall. Most of the discussion section reads like it should be in the results section. | Thank you for providing this feedback. A number of changes have been made to the results and discussion section, and the additon of tables improves readability. |  |  | 10 | 314 |
| 2 | Results:    I think this is a good place to show which studies explored which types of interventions as well. | Thank you for this comment. An exploration of which studies explored which interventions is below the 'quality of included studies' section. We have discussed this and would like to keep the order of these subheadings unaltered, please see below for a rationale. | No change | No change | 10 | 314 |
| 2 | Results:  I don’t think there is a need to still describe which studies outside of the overview of the study. For instance, under CBT- based groups, the authors still describe the studies covered there. I think this is information that should be included under the overview of the study. | Thank you very much for this feedback. We have discussed the order of the result section and feel that it is important to the overall interpretation of the results to know that the majority of studies were weak in quality. Therefore we have decided to present an overview of the quality assessment first, to caveat the description of individual studies, so that readers are aware that not all findings were rigorous. Both the quality assessment and the description of the studies are important aspects of the overview. | No change | No change | 10 | 314 |
| 2 | -       Kindly appraise each included study (report percentages as well). | Thank you for this comment. Tables 2, 3 and 4 provide details of how weakness was assessed using the tools. These tables are now attached. |  | See tables | 11 | 341 |
| 2 | -       May the authors please include a table showing the characteristics of the studies selected? This would be what is under overview of studies but in detail. In the package I received for review, there was no Table 1, the only attachments I saw was the checklist and the prisma diagram. | Thank you for this useful feedback. The tables were not provided in error, but they are now included and provide an overview of the study characteristics. |  | See tables | 11 | 341 |
| 2 | o   Authors can include authors, country, design, sample, core findings and limitations. This gives readers a better understanding of the review and studies included. | Thank you for this helpful feedback. This is included in Table 1. |  | See tables | 11 | 341 |
| 2 | Results:    Under the overview of the study, I suggest the authors report these in a different manner. It reads as if meta-inference was done, and this was not previously proposed. For example, “The average sample size was 199 participants ranging from 15 participants…” this alludes that the authors analyzed the studies to attain averages etc. To do this, the authors would need a more homogeneous study sample which was not a priority (referring to the inclusion and exclusion criteria). Perhaps consider a table that will outline the desired items the authors want to highlight from your study that adds impact to your study. | Thank you for your detailed comments, and useful suggestion of a table. Tables 1-4 are now attached. To clarify, meta-interference was not done. Averages are reported to give an overview of the characteristics of the studies in a concise manner. |  | See tables | 11 | 341 |
| 2 | Results:      I suggest that the authors double check their study references, for instance when they say “Seven studies directly measured exposure to traumatic events…” but the referenced studies are eight. | Thank you for this useful feedback. This has now been changed. The missed study was the one which was identified through citation searching and therefore did not appear in an earlier draft. All references have been checked for accuracy. | Seven studies directly measured exposure to traumatic events (19,20,23,28,33,38,42,45). | **Eight** studies directly measured exposure to traumatic events [23,26,28,30,32,38,41,43]. | 21 | 346 |
| 1 | Line 185 Put this in a table formart for the reader to understand. On the table, unpack scope of research, publication date, analysis, field, etc. | Thank you for this comment. Tables 1-4 are now attached and provide the details you have mentioned. |  | See tables | 22 | 386 |
| 2 | **Quality of included studies:**  Please refer to comment on appraisal. It is still a concern that the authors continuously mention the weakness of the selected study; this ultimately reduces the reliability of your own review. I would also suggest that this comes right after the results heading. | Thank you for these comments. Tables 2, 3 and 4 provide details of how weakness was assessed using the tools. These tables are now attached. |  | See tables | 22 | 386 |
| 2 | -       It is important to have a method of analysis where the authors analyze the studies and report accordingly. This proposes an issue in the result section when authors outline one or two studies and as a reader we are wondering about the other studies and why they were not reported. There is a need to collate this studies and not only describe them (this is no different to the overview of the study). | Thank you for your feedback. All 25 studies included in the review are summarised in the results section, this is done thematically by type of intervention. Studies which were unique in their intervention type are reported singularly towards the end of the results section. The tables are now included, which makes the summary of included studies easier to interpret. |  | See tables | 22 | 386 |
| Editor | 5. Please include a copy of Tables 1-4 which you refer to in your text on pages 8 and 9. | Thank you for this comment. Tables 1-4 are now attached. |  | See tables | 22 | 386 |
| Editor | 2. Thank you for stating the following financial disclosure: “This work was supported by the National Centre for Population Health and Wellbeing Research. Funding was provided by Public Health Wales and the Economic and Social Research Council (ESRC) through their suport of a PhD studentship” Please state what role the funders took in the study. If the funders had no role, please state: "The funders had no role in study design, data collection and analysis, decision to publish, or preparation of the manuscript." If this statement is not correct you must amend it as needed. Please include this amended Role of Funder statement in your cover letter; we will change the online submission form on your behalf. | Thank you for this comment. The statement has been updated accordingly. | This work was supported by the National Centre for Population Health and Wellbeing Research. Funding was provided by Public Health Wales and the Economic and Social Research Council (ESRC) through their suport of a PhD studentship | This work was supported by the National Centre for Population Health and Wellbeing Research. Funding was provided by Public Health Wales and the Economic and Social Research Council (ESRC) through their suport of a PhD studentship. **The funders had no role in study design, data collection and analysis, decision to publish, or preparation of the manuscript.** | 38 | 849 |
| Editor | 1. Please ensure that your manuscript meets PLOS ONE's style requirements, including those for file naming. The PLOS ONE style templates can be found at https://journals.plos.org/plosone/s/file?id=wjVg/PLOSOne_formatting_sample_main_body.pdf and https://journals.plos.org/plosone/s/file?id=ba62/PLOSOne_formatting_sample_title_authors_affiliations.pdf | Thank you for this comment. Headings and subheadings have now been amended to meet formatting guidelines. |  |  |  |  |
| Editor | 1. Please ensure that your manuscript meets PLOS ONE's style requirements, including those for file naming. The PLOS ONE style templates can be found at https://journals.plos.org/plosone/s/file?id=wjVg/PLOSOne_formatting_sample_main_body.pdf and https://journals.plos.org/plosone/s/file?id=ba62/PLOSOne_formatting_sample_title_authors_affiliations.pdf | Thank you for this comment. Fig1 is now available as .TIFF. This has also been through PACE to ensure dimensions are correct. |  |  |  |  |
| Editor | 3. Thank you for uploading your study's underlying data set. Unfortunately, the repository you have noted in your Data Availability statement does not qualify as an acceptable data repository according to PLOS's standards. At this time, please upload the minimal data set necessary to replicate your study's findings to a stable, public repository (such as figshare or Dryad) and provide us with the relevant URLs, DOIs, or accession numbers that may be used to access these data. For a list of recommended repositories and additional information on PLOS standards for data deposition, please see https://journals.plos.org/plosone/s/recommended-repositories. | Thank you for providing this guidance. The underlying dataset (search strategy and summaries of included studies and quality assurance) are now uploaded to Dryad. Please note the search strategy is provided under 'supplementary information'. These can be accessed at https://datadryad.org/stash/share/fGnZm4C2ZwqkLjNu9zX-iWAMtZdYTfv9qUMeSetSn7o |  |  |  |  |
| Editor | 4. Please include a separate caption for each figure in your manuscript. | Thank you for your feedback. This has now been done. There is only one figure in this manuscript which is the PRISMA diagram, this has been provided as a separate .TIFF file |  |  |  |  |
| 1 | Referencing: Is this page numbers or sources? if is a source, which one? | Thank you for your comments on referencing. The manuscript uses PLOS ONE/Vancouver style referencing. The reference list appears in the order the works are cited, and the numbers in square brackets in the text denote this. The PLOS One guidelines state: "In the text, cite the reference number in square brackets (e.g. "We used the techniques developed by our colleagues [19] to analyse the data")." Using author names in some places but not others is consistent with the PLOS One referencing guidelines. The choice to use author names in some places is based on sentence structure and the manuscript being as easy to read as possible. |  |  |  |  |
| 1 | Line 164 referencing Be consistent with referencing, if you include numbers only with no names of authors, it has to be clear. From introduction page, you only used numbers and no names of authors, on page 4, you included numbers and names of authors.  how do you want to reference in this papers? |  |  |  |  |  |
| 1 | Line 328 Be consistent with referencing please, see previous comments |  |  |  |  |  |
| 1 | Line 333 See the difference on your referencing style… |  |  |  |  |  |
| 1 | Line 333 Why did you use name and number |  |  |  |  |  |
| 1 | Line 356 ???? this is not consistent.. |  |  |  |  |  |
| 2 | -       There is a need for the authors to attend to document punctuation and English editing for readability. | Thank you for this comment. A number of edits have been made to remove typos and to improve the readablity of the document. |  |  |  |  |
| 2 | I think the introduction needs a bit more work to build up the review. | Thank you for this feedback. A number of edits have been made to the introduction to improve overall structure and readability. |  |  |  |  |
| 2 | -       The prisma diagram is not clear to read. | Thank you for this useful feedback, the PRISMA diagram has been re-exported as a TIFF file. |  |  |  |  |
