# Supplementary material for: A systematic review of non-clinician trauma-based interventions for school-age youth
Source: PLoS One. 2024 Sep 6;19(9):e0293248. doi: 10.1371/journal.pone.0293248 (PMC11379276; doi:10.1371/journal.pone.0293248)
Supplement: Attachment — Submitted filename: PLOSONE Reviewer comments2.docx [file pone.0293248.s008.docx]

**Reviewer comments.**

Thank you to the authors for all the work they have put into the manuscript. As initially stated, this is a very good idea for a review and will be a good addition to the body of knowledge.

I appreciate and acknowledge the changes made to the review and the explanations for maintaining some parts as originally stated. However, I have a few more comments for your attention.

**Abstract.**

Line no 32: For clarity, please state the search bracket in full: from January 2013 till when?

Line 52-53: I don’t believe that’s a key finding to a review; line 50 can serve as a key finding. Remember, your review does not assess the quality of studies but the efficacy of non-clinician intervention. A key finding should be something that answers your research question best. Unless you are saying that the interventions themselves were of low quality and, therefore, the desired results are rarely achieved.

**Introduction**

Line 71-71, first paragraph: I think it will benefit the readers to state at least one or two combinations of ACEs that resulted in trauma. I am suggesting this because it seems like there is a gap; you say that experiencing an ACE does not result in trauma experience; however, a combination of some ACEs can; as a reader, I’m thinking if an ACE does not equal trauma but a marriage of a couple of them does, what’s that marriage consisting of? You don’t want a reader to pause and search “ which combination of ACEs causes trauma” but rather search for a gap from your overall study.

**Study selection**

Authors should include a breakdown of which databases were used, how data were searched, and which database yielded how many results; at the end, how many articles were included from each database in the final review? The scientific aspect of a systematic review is that it can be replicated. However, a thorough description of the methodology and selection should be outlined.

**Analysis and discussion**

There is still a need for clarity on how data was analyzed. How did you synthesize information from different articles?

There is confusion about the study's aim. Are you assessing the quality of studies or the effectiveness of non-clinical methods that attend to trauma caused by ACEs? There is a continuous mention of the study quality (which should only be reported in the appraisal, not as part of a finding). For me, the discussion still reads as the study findings; the authors are not discussing the findings but re-presenting the findings.
