# Supplementary material for: A systematic review of non-clinician trauma-based interventions for school-age youth
Source: PLoS One. 2024 Sep 6;19(9):e0293248. doi: 10.1371/journal.pone.0293248 (PMC11379276; doi:10.1371/journal.pone.0293248)
Supplement: Attachment — Submitted filename: 2.0 Response to Reviewers Word.docx [file pone.0293248.s009.docx]

| **Reviewer comments** | **Response** |
| --- | --- |
| Thank you to the authors for all the work they have put into the manuscript. As initially stated, this is a very good idea for a review and will be a good addition to the body of knowledge.  I appreciate and acknowledge the changes made to the review and the explanations for maintaining some parts as originally stated. However, I have a few more comments for your attention. | Thank you very much for your positive feedback. |
| Line no 32: For clarity, please state the search bracket in full: from January 2013 till when? | Thank you, the following amendment has been made:   - Line 27: This has been changed to ‘between January 2013 and April 2023’ - Line 113: changed to state ‘1^st^ January 2023’ - Please see appendix for exact dates of the searches (which give exact end dates for the search bracket) |
| Abstract Line 52-53: I don’t believe that’s a key finding to a review; line 50 can serve as a key finding. Remember, your review does not assess the quality of studies but the efficacy of non-clinician intervention. A key finding should be something that answers your research question best. Unless you are saying that the interventions themselves were of low quality and, therefore, the desired results are rarely achieved. | Thank you for this detailed and helpful feedback. This has now been edited:   - Line 34: A key finding was that there is a lack of high-quality research evidence to inform non-clinician delivered trauma-informed interventions. Many included studies were weak quality due to convenience sampling of participants and potential bias. |
| **Introduction**  Line 71-71, first paragraph: I think it will benefit the readers to state at least one or two combinations of ACEs that resulted in trauma. I am suggesting this because it seems like there is a gap; you say that experiencing an ACE does not result in trauma experience; however, a combination of some ACEs can; as a reader, I’m thinking if an ACE does not equal trauma but a marriage of a couple of them does, what’s that marriage consisting of? You don’t want a reader to pause and search “ which combination of ACEs causes trauma” but rather search for a gap from your overall study. | Thank you for this comment and for your detailed explanation. I agree I worded this section in a confusing way and I have now amended. There is a relationship between having just one ACE and trauma symptoms (this makes sense if you think about ACEs like sexual abuse and physical abuse). I have added a reference for this. Furthermore, the relationship between ACEs and trauma is stronger for multiple ACEs (but the relationship is there for any number of ACEs). I have amended the wording to clarify this:   - Line 52: While not everyone who has ACEs necessarily experiences a traumatic event [4], an ACE score of 1 or more is associated with the development of trauma symptoms [5], and this relationship is even stronger where four or more ACEs have occurred [6]. |
| **Study selection**  Authors should include a breakdown of which databases were used, how data were searched, and which database yielded how many results; at the end, how many articles were included from each database in the final review? The scientific aspect of a systematic review is that it can be replicated. However, a thorough description of the methodology and selection should be outlined. | Thank you for your comment. I am wondering if you received appendix 1 with the most recent manuscript – this includes a detailed search strategy which states ‘which databases were used, how data were searched, and which database yielded how many results’. Please can I ask if there was an issue and this was missing? |
| **Analysis and discussion**  There is still a need for clarity on how data was analyzed. How did you synthesize information from different articles? | Thank you for this comment. I have added a sentence:   - Line 214: Information from all articles was extracted into a data extraction table on Excel so that included studies could be compared and analysed.   Line 117 details who extracted the data and who checked this. I have also added a line here:   - Data extraction headings can be found in Appendix 2.   Appendix 2 has now been created and attached. |
| There is confusion about the study's aim. Are you assessing the quality of studies or the effectiveness of non-clinical methods that attend to trauma caused by ACEs? There is a continuous mention of the study quality (which should only be reported in the appraisal, not as part of a finding). For me, the discussion still reads as the study findings; the authors are not discussing the findings but re-presenting the findings. | Thank you for your comments. The aim is to assess the effectiveness of interventions. There is little good evidence for the effectiveness of available interventions due to the study quality. A change has been made to the discussion section to reflect this.   - Line 529: Overall, the evidence base for effectiveness of trauma recovery interventions was found to be weak, due to the quality of the studies addressing this research area. |
