# Supplementary material for: A systematic review of non-clinician trauma-based interventions for school-age youth
Source: PLoS One. 2024 Sep 6;19(9):e0293248. doi: 10.1371/journal.pone.0293248 (PMC11379276; doi:10.1371/journal.pone.0293248)
Supplement: Attachment — Submitted filename: 3.0 Response to reviewers.docx [file pone.0293248.s010.docx]

Response to reviewers

|  | Comment | My response (all line references are for the track changes version) |
| --- | --- | --- |
| 3 | My main concern is the balance of information presented in the main article vs the appendix. I think the authors would benefit from comparing their manuscript with other published systematic reviews in this journal. For example, it is unusual to see tables breaking down the scores for quality assessment for each item in those checklists in the main paper - this takes up a lot of room which would be better served with more information about your search strategy. Instead, tables 2-4 should be in your appendix, with the overall ratings added to Table 1. | Thank you for this comment. I have reorganised the supporting information for clarity and moved the tables as suggested.  Supporting information: 1: PRISMA diagram  2: Characteristics of included studies  3: Quality assessment tables  4: Search strategy  5: Data extraction template |
| 3 | Whilst you have completed a PRISMA diagram, this currently isn't referenced anywhere in the text. Figure 1 should therefore be signposted at the beginning of your results. | Added at line 212 and 275. The PRISMA diagram can be found in SI 1. |
| 3 | it doesn't make sense to include the information about data extraction in the 'review design' section (lines 98-100), especially as it is detailed in the 'study selection' section below - suggest removing. | This has been amended at line 144, and the sentence ‘Data extraction headings can be found in Supporting Information 5’ has been moved to the study selection section. (line 222) |
| 3 | language of publication was restricted to which languages? This needs to be specified | restricted to English – this has been added at line 180. |
| 3 | lines 163-165 - you do not need to repeat that this paper was included or need the detail of it being uploaded to Covidence - this is too much detail for a paper. This can be removed - stating "resulting in the identification of one additional paper" is sufficient. | Thank you, a previous reviewer had asked for further detail. I agree with your comments and this has now been deleted (at line 226) |
| 3 | the new text on line 192 should be in the methods, not the results. | The line ‘Information from all articles was extracted into a data extraction table on Excel so that included studies could be compared and analysed’ has been moved to the methods section (Line 217). |
|  | You need to have a section entitled 'data synthesis' in your methods section. This should also explain your approach to data synthesis e.g. did you do a narrative synthesis? | This has been added. (Line 259) |
| 3 | overall, I felt like the overall importance of implications of this review are understated - both in the introduction and discussion. You point out several limitations and gaps in existing literature, but the discussion would benefits from including more specific examples of novel interventions or trials which could be conducted. Think about answering the question "so what?" | I have added further details to the discussion session. |
| 4 | Authors should identify synthesis methods in the abstract. | Thank you, this has been added at line 33 |
| 4 | The sentence beginning on line 43 is confusing “While not everyone who has ACEs necessarily experiences a traumatic event [4], an ACE score of 1 or more is associated with the development of trauma symptoms [5], and this relationship is even stronger where four or more ACEs have occurred [6].” I think by definition if you experience an ACE you experience a traumatic event. The previous sentence already outlines that 4+ ACEs is more strongly associated with trauma symptoms. I would suggest amending this sentence for clarity or deleting. | This sentence was added following feedback from a previous reviewer, I agree with your comments and I have removed it (Line 50) |
| 4 | The authors go on in the second paragraph to discuss the importance of increasing ACE awareness. It is unclear if they are referring to ACE-related trauma symptoms or ACE incidence. | Thank you for this, I have updated this sentence to clarify. “Promoting awareness of ACEs and the potentially traumatic effects of these experiences …” at line 55 |
|  | Further, the suggestion of trauma-informed services being recommended is not linked to a reason why, for what have they been recommended? I suggest editing this section for clarity. | This paragraph has been amended (Line 57) |
| 4 | The authors refer to the target population as “youth with trauma” in line 59, I would suggest a more appropriate way to describe the population here would be ‘youth who have experienced potentially traumatic events’ or youth experiencing trauma-related symptoms – depending on the populations represented in the reviews being referred to. Throughout the review this is an important distinction to clarify – are you referring to universal interventions for any youth who has been exposed to potentially traumatic events or those experiencing trauma-related symptoms? | Thank you for this comment. This review is primarily aimed at developing an evidence base for universal trauma-based interventions which can be used with young people with exposure to ACEs, or who are likely to have exposure to ACEs. Disclosure of traumatic experiences or a diagnosis of PTSD are not prerequisites for inclusion in the review, as practically for a non-clinician to deliver an intervention in a school setting you would not ask for this information. Although many included studies did use PTSD as their inclusion criteria, not all did and it is not a necessary feature.  I have reviewed the manuscript in full and removed references to ‘youth with trauma’ or similar, and replaced it with ‘young people with exposure to ACEs’ or similar. (see Line 93, 579, 590, 598, 643, 696) |
| 4 | The review would benefit from more clarity around the research question. Are the authors interested in universal prevention of trauma symptoms in youth with any exposure to ACEs, or targeted interventions for youth experiencing trauma symptoms after exposure to ACEs? | This review concerns universal support in youth with any exposure to ACEs. The introduction has been amended to clarify this. |
| 4 | The research question “what evidence exists for the efficacy of non-clinician delivered interventions for supporting trauma recovery or improvements in mental health in school-age youth (4-18 years) who have experienced ACEs?” suggests the authors’ target population are youth experiencing trauma symptoms (who were exposed to ACEs) and the inclusion criteria state indicate they looked for articles describing interventions targeting ‘recovery’ however they have not extracted data to indicate symptom severity. This is an important factor when reporting efficacy in reducing symptoms across different studies and interventions. Without giving the reader, the participants’ baseline trauma symptom severity or level of ACE exposure, it is difficult to compare the interventions. | Although many of the studies did look at PTSD symptoms, this was not a prerequisite for inclusion in the review. This has been changed throughout to reference potentially traumatic events and mental health.  Not all studies recorded PTSD symptoms and there was heterogeneity in the measures used amongst those that did. Therefore we have decided to not extract trauma symptom severity. |
|  | In the characteristics of studies table, some studies describe the measure for trauma/PTSD as “at least one ACE” – over 60% of the population have experienced at least one ACE. Comparing efficacy of interventions in populations with one ACE against participants with over 4 ACEs is very different, the authors should provide information on trauma symptom severity and take baseline severity into account when making suggestions on which interventions may be effective for which population groups. Alternatively, the authors could divide into two research questions – one addressing universal prevention interventions for youth exposed to ACEs and another for youth with trauma-related symptoms. | All included studies either recruited the study population based on likely exposure to trauma, measured exposure to traumatic events, or measured PTSD symptoms (this is stated in the overview of studies).  Supporting Information 2. Characteristics of included studies – this has been updated to make it explicit that all populations had some level of ACE exposure or PTSD or both. |
| 4 | I have concerns that the search strategy was not aligned well with the research question and was potentially not comprehensive enough to provide a true overview of the evidence in the field. The authors state the target population is youth who have experienced ACEs, yet they have not included ACE-related search terms. I am concerned they may have missed relevant articles by not including these terms. If it is the authors intention to include articles with any ACE exposure but not required to be experiencing trauma-related symptoms, I suggest they re-run the searches with inclusion of ACE-related terms and terms for each ACE category (e.g. abuse, violence, neglect, maltreatment, assault, divorce, incarceration etc) to include studies that have assessed the efficacy of interventions for youth exposed to any ACEs. The trauma search terms are very limited, and studies may have been missed that use terms such as ‘distress’ or ‘posttraumatic’ or ‘mental health’. | Initial scoping searches indicated that there was a lack of studies in this area which targeted the impact of specific ACEs such as domestic violence, and so the decision was made to broaden it out to be more universal and aimed at ACEs in general.  The authors have taken the decision to not re-run this search. Adding the suggested terms to WoS alone takes it from 2,105 results to 15,500. This is not sustainable and beyond the scope of this review, especially given that scoping searches did not indicate this would result in more useful studies.  A section has been added under limitations to suggest a wider review as a possibility for future research (line 680) |
| 4 | The data extracted should include sample size and information on how long and often it was implemented e.g. length of intervention, number of sessions, length of sessions, frequency and who delivered – this is one of the key characteristics of the review – the non-clinician delivery, therefore the reader is interested in what type of non-clinician delivered the intervention. | Thank you for this comment. I have added this to the data extraction table and put it in the Supplementary Information.  Sample size and length and frequency information is now added. |
| 4 | The purpose in the characteristics of studies should have the comparator added for RCTs, it would also be helpful for the reader if table 1 included sample size and intervention setting, severity of symptoms/no. of ACEs and mode of delivery. | The sample size has been added. Mode of delivery has been added for the three online interventions, all others were in person delivery. There is not space in the table to add the other requested information. The ‘measure for ACEs or trauma’ column has been updated to make explicit that all samples were high ACEs or had trauma symptoms measured. |
| 4 | The paragraph starting on line 208 mixes mode of delivery (e.g. online) with intervention setting (e.g. school) – these should be described separately. Does residential setting mean in the participants home? If so, this would be a more informative description. | This information has now been added at line 288. |
| 4 | Line 235 introduces a paragraph describing the interventions assessed by the nine studies with moderate or strong quality evidence but only 8 are then listed. | Thank you for this, I have added the missing references at line 317 |
| 4 | Note RCT is an acronym, so the correct phrase is a RCT rather than an RCT. | Thank you for this comment, this has been amended on line 358 |
| 4 | Line 256 – “it is suggested..” do the study authors suggest this or the review authors – suggest clarifying | Thank you for this comment, this line has been amended to read ‘the authors suggest’ Line 333 |
| 4 | In the paragraph reporting results from Elswick et al. the authors do not provide any information of the efficacy of the intervention. | This has now been added at Line 377 |
| 4 | The overview of the current evidence and gaps in the field is incomplete. Several publications have reviewed school-based interventions for trauma symptoms. Reference to these could be useful to highlight the current article’s contribution to the literature e.g. Stratford et al 2020, Berger et al 2019, Chafouleas et al 2019. | The introduction currently references Lange et al. 2022 and Marie-Mitchell et al. 2019, in making the point that many existing reviews have addressed school-based interventions to support with trauma, but have mainly focused on clinician-delivered interventions. I have added Chafoules to this section as this paper provides a similar overview, thank you for sharing this (referenced line 83).  Stratford is already referenced as well as a key finding of this review was that non-clinician interventions warrant further research.  I have reviewed the Berger paper, this paper is mainly focused on multi-tiered approaches which is not as relevant to the present study and the rationale in the introduction so we have decided to not include this, although it has now been referenced in a relevant part of the discussion (line 666). |
| 4 | The discussion is restating much of the results and should be revised to avoid re-presenting results but providing interpretation and comparison to existing literature and highlighting what the review add to the field. While the authors attempt to state whether the research question was answered, this is structured as though the question was to assess the quality of evidence followed by presentation of results. The discussion should be re-written so as to provide the reader with clarity on how the reviews finding answer the question and how this relates to existing evidence. | I have added further details to the discussion session. |
