# Supplementary material for: A systematic review of non-clinician trauma-based interventions for school-age youth
Source: PLoS One. 2024 Sep 6;19(9):e0293248. doi: 10.1371/journal.pone.0293248 (PMC11379276; doi:10.1371/journal.pone.0293248)
Supplement: Attachment — Submitted filename: PLOSONE Reviewer comments.docx [file pone.0293248.s006.docx]

Reviewer comments:

**General.**

I believe this is a good review topic to consider and a very necessary one at that. However, the review still needs quite a lot of work (major revisions). Please see comments below.

- There is a need for the authors to attend to document punctuation and English editing for readability.
- To reduce confusion, I suggest that the title reads “… intervention for school-aged youth.” As this is clearly stated as such within the paper. I would also suggest that the authors choose how they will describe their population of interest and be consistent. Should they choose to use school-aged youth for instance, that should be throughout the whole paper.

**Abstract.**

- Line 22, where is this happening?
- What was the review question?
- Which search engines did you use?
- Which keywords or phrases did the authors use to search for data?
- Which appraisal tool did you use?
- How did you analyze your data?
- Some of your leading selection criteria guides?
- Line 32 and 33, what does “weak quality, and high quality” imply?
- What are the findings of your review? From line 31-35 read as limitations of this review, line 35- 37 read as a recommendation.
- I believe answering these questions will make the abstract more comprehensive.

**Introduction.**

- May the authors kindly reference statement 46-48.
- Line 52, which ACEs result in the development of trauma when experienced concurrently?
- In the context which the authours are basing the review, what’s the status on ACE related trauma and counselling (both formal and informal)?
- Line 64, where was this?
- Line 66, “ a more population approach is needed” what does that mean? Could this be rephased for clarity?
- It would be good to have context to your review and read about the available non- clinician rehabilitation or support prior to bringing us into the assessment of these approaches. What kind of programmes / tools are they? Who were they designed for (demographically), who administrates them?
- I think the introduction needs a bit more work to build up the review.
- Line 74, this research question tells us that the authors aim to look for the tools and their effectiveness. However, your abstracts only alludes to assessing the effectiveness hence one of my comments was asking about the available tools. It is important for the authors to be clearer with what they aim to do in the review and eliminate ambiguity.

**Methods**

- Line 106 adolescent is incomplete.
- I think the last line 122-123 should be removed because that is already obvious from the 10 years section criteria mentioned. What this does is only bring up the question “ why 10 years?” which I would understand because interventions implemented for a longer period can easily show effectiveness as they are assessed overtime and may possibly be applied on different demographics.
- Was this a world wide review?
- In terms of your inclusion and exclusion criteria, the duration of intervention implementation; doesn’t that count?
- The prisma diagram is not clear to read.
  - Studies screened, what type of screening was that?
    - Studies excluded (n=3770), on what bases were they excluded?
    - Kindly show when title, abstract and full screening was done and categorize reasons for exclusion accordingly (as stated in line 147)
  - May the authors please include that they also searched for studies via citation under study selection.
- Kindly appraise each included study (report percentages as well).
- May the authors please include a table showing the characteristics of the studies selected? This would be what is under overview of studies but in detail. In the package I received for review, there was no Table 1, the only attachments I saw was the checklist and the prisma diagram.
  - Authors can include authors, country, design, sample, core findings and limitations. This gives readers a better understanding of the review and studies included.
- Method of analysis is missing.

**Results:**

- Under the overview of the study, I suggest the authors report these in a different manner. It reads as if meta-inference was done, and this was not previously proposed. For example, “The average sample size was 199 participants ranging from 15 participants…” this alludes that the authors analyzed the studies to attain averages etc. To do this, the authors would need a more homogeneous study sample which was not a priority (referring to the inclusion and exclusion criteria). Perhaps consider a table that will outline the desired items the authors want to highlight from your study that adds impact to your study.
- I suggest that the authors double check their study references, for instance when they say “Seven studies directly measured exposure to traumatic events…” but the referenced studies are eight.
- I think this is a good place to show which studies explored which types of interventions as well.
- I don’t think there is a need to still describe which studies outside of the overview of the study. For instance, under CBT- based groups, the authors still describe the studies covered there. I think this is information that should be included under the overview of the study.

**Quality of included studies:**  Please refer to comment on appraisal. It is still a concern that the authors continuously mention the weakness of the selected study; this ultimately reduces the reliability of your own review. I would also suggest that this comes right after the results heading.

- It is important to have a method of analysis where the authors analyze the studies and report accordingly. This proposes an issue in the result section when authors outline one or two studies and as a reader we are wondering about the other studies and why they were not reported. There is a need to collate this studies and not only describe them (this is no different to the overview of the study).
- The results and discussion section need to be reworked overall. Most of the discussion section reads like it should be in the results section.
